# The identification of a TNBC liver metastasis gene signature by sequential CTC‐xenograft modeling

**DOI:** 10.1002/1878-0261.12533

**Published:** 2019-06-19

**Authors:** Monika Vishnoi, Nikki Haowen Liu, Wei Yin, Debasish Boral, Antonio Scamardo, David Hong, Dario Marchetti

**Affiliations:** ^1^ Biomarker Research Program Center Houston Methodist Research Institute TX USA; ^2^ Department of Investigational Cancer Therapeutics The University of Texas MD Anderson Cancer Center Houston TX USA

**Keywords:** bone marrow‐resident tumor cells, circulating tumor cells, CTC‐derived xenografts, Genomics/transcriptomic analyses of CDX‐derived CTC populations, liver metastasis, triple‐negative breast cancer

## Abstract

Triple‐negative breast cancer (TNBC) liver metastasis is associated with poor prognosis and low patient survival. It occurs when tumor cells disseminate from primary tumors, circulate in blood/lymph [circulating tumor cells (CTCs)], and acquire distinct characteristics during disease progression toward the metastatic phenotype. The purpose of this study was to decipher the genomic/transcriptomic properties of TNBC liver metastasis and its recurrence for potential therapeutic targeting. We employed a negative depletion strategy to isolate and interrogate CTCs from the blood of patients with TNBC, and to establish sequential generations of CTC‐derived xenografts (CDXs) through injection of patient CTCs in immunodeficient mice. The isolation and validation of CDX‐derived cell populations [analyses of CTCs were paired with bone marrow‐resident cells (BMRTCs) and liver tissue cells obtained from the same animal] were performed by multiparametric flow cytometry, immune phenotyping, and genomic sequencing of putative CTCs. Comprehensive characterization of gene expression arrays from sequentially generated CDX‐derived cell populations, online gene expression arrays, and TCGA databases were employed to discover a CTC‐driven, liver metastasis‐associated TNBC signature. We discovered a distinct transcriptomic signature of TNBC patient‐isolated CTCs from primary TNBCs, which was consistent throughout sequential CDX modeling. We established a novel TNBC liver metastasis‐specific CDX model that selectively recapitulates CTC biology for four sequential generations of mice. The evaluation of online databases and CDX‐derived populations revealed 597 genes specific to the TNBC liver metastasis signatures. Further investigation of the TNBC liver metastasis signature predicted 16 hub genes, 6 biomarkers with clinically available drugs, and 22 survival genes. The sequential interrogation of CDX‐CTCs is an innovative liquid biopsy‐based approach for the discovery of organ metastasis‐specific signatures of CTCs. This represents the first step for mechanistic and analytical validation in their application as prognostic indicators and therapeutic targets. Targeting CTC drug candidate biomarkers along with combination therapy can improve the clinical outcome of TNBC patients in general and recurrence of liver metastasis in particular.

**Abbreviations**
BM: bone marrow
BMRTC: bone marrow‐resident tumor cell
CDX: CTC‐derived xenograft
CK: cytokeratin
CTC: circulating tumor cell
DGE: differential gene expression analysis
EpCAM: epithelial cell adhesion molecule
FC: fold change
FDA: Federal Drug Administration
GCDFP‐15: gross cystic disease fluid protein‐15
H&E: hematoxylin & eosin
HCC: hepatocellular carcinoma
NAT: normal adjacent tissue
OS: overall survival
PBMC: peripheral blood mononuclear cell
pTNBCs: primary triple‐negative breast cancer tissues
TNBC: Triple‐negative breast cancer

## 1 Introduction

Triple‐negative breast cancer (TNBC) is the most aggressive form of breast cancer with poor therapeutic options. It is a subtype characterized by the absence of hormone receptor (ER/PR) expression and HER‐2 expression/amplification (Boyle, 2012). TNBC associates with worst prognosis and distant‐free survival vs other non‐TNBC breast cancer subtypes (Agarwal *et al*., 2016). Liver is one of the most common organ sites of TNBC metastasis in cohorts associated with worst prognosis and survival. Approximately 10% of TNBC patients are diagnosed with liver metastasis; however, this percentage rises up to 25% when combined with other visceral organ metastasis (Agarwal *et al*., 2016; Boyle, 2012). Despite extensive chemotherapy, immunotherapy, and/or their combination, stage IV TNBC patients with liver metastasis have a median survival time of only 12 months (Al‐Mahmood *et al*., 2018; Wang *et al*., 2017). Therefore, early prognosis and detection of liver metastasis is imperative to improve the life expectancy of TNBC patients.

Breast cancer liver metastasis results from circulating tumor cells (CTCs) shed from primary and/or metastatic tumors, their dissemination in the circulation, along with CTC transit and prolong residence in bone marrow‐resident tumor cells (BMRTCs). CTC/BMRTC subsets are thus ‘seed of metastasis’, migrate to distant organs, attain capacities to self‐renewal, and develop metastasis in multiple organ microenvironments, including liver (Alix‐Panabieres *et al*., 2008; Joosse *et al*., 2015; Muller *et al*., 2010; Pantel and Alix‐Panabieres, 2019; Paoletti and Hayes, 2016; Riethdorf *et al*., 2008). CTC/BMRTC acquiring genomic and/or molecular properties are distinct from ones of primary tumors and capable of fostering metastatic potency (Boral *et al*., 2017; Paget, 1889). For example, the presence of CTCs in TNBC patients was found to be an independent prognostic indicator associated with poor survival (Hayes and Smerage, 2008; Lu *et al*., 2016; Paoletti *et al*., 2015). CTCs are rare, highly heterogeneous, and ER/PR, prognostic EGFR, and HER‐2 expression levels are discordant with patient diagnosis or disease stage (Agelaki *et al*., 2017). In TNBC patients, prognosis of CTCs and natural killer cells is clinically relevant and associated with progression‐free survival (Liu *et al*., 2018a). The molecular characterization of CTCs/BMRTCs with metastasis‐initiating capabilities can therefore reveal prognostic and diagnostic biomarkers, which are critical not only for an improved understanding of the development of metastasis but also as determinants for early‐stage TNBC and its progression (Pantel and Alix‐Panabieres, 2019; Powell *et al*., 2012; Sprouse *et al*., 2019).

In the era of precision medicine, real‐time monitoring of disease progression is imperative. Here, we generated TNBC‐CTC‐derived xenografts (CDXs). These CDX models are highly relevant since they are able to faithfully recapitulate the biology of disease progression. They can also provide fundamental advances in the biology of patient‐derived CTC dissemination, survival in the circulation, and CTC transitions from dormancy to metastasis‐competent states during progression (Kang and Pantel, 2013). We have recently demonstrated that CTCs home to and reside in BM of CDX models during the asymptomatic phase of disease, along with the discovery of a metastasis‐competent BMRTC state (Vishnoi *et al*., 2018).

We hypothesized that liver metastasis in TNBC patients arises from a subset of a heterogeneous CTC population possessing properties distinct from the primary tumor during the selective and rate‐limiting steps of tumor progression over time. To identify a TNBC liver metastasis transcriptional signature consistently associated with CTC, we demonstrated the development and interrogation of a CDX model of TNBC liver metastasis to successfully recapitulate CTC dissemination abilities associated with liver metastasis in sequential CDX generations (four). Furthermore, we identified a CDX transcriptomic signature (597 genes), found to be present in paired CDX populations (CDX‐CTCs, BMRTCs, and metastatic liver cells obtained from the same animal), by injecting *de novo* CTCs isolated from TNBC patients and their longitudinal monitoring in CDX generations.

## 2 Methods

### 2.1 Human subjects

Patients diagnosed with TNBC were accrued according to a protocol approved by Institutional Ethical Review Board at MD Anderson Cancer Center (MDACC) and Houston Methodist Research Institute (HMRI). Patient blood samples (30–35 mls) were collected after receiving a written consent from the patient and according to the principles of the Declaration of Helsinki. Clinical details and parameters for each patient are provided in Table S1. All samples were collected in EDTA tubes and provided immediately to the laboratory for CTC isolation and analysis.

### 2.2 CTC enumeration and CellSearch™

Peripheral blood mononuclear cells (PBMCs) were isolated using red blood cell lysis buffer (154 mm NH_4_Cl, 10 mm KHCO_3_, and 0.1 mm EDTA) either from patient blood (8 mL), CDX blood (500‐800 μL), or BM (Vishnoi *et al*., 2018). PBMCs were stained with antibodies for respective markers and loaded to FACSAria III flow cytometer (BD Biosciences, San Jose, CA, USA) for multiparametric selection. All sorted‐cell population were collected in 10% Dulbecco's modified Eagle's medium‐F12 media + 90% 1× PBS (both from Gibco, Dún Laoghaire, Dublin, Ireland) for downstream interrogation. FACS data were analyzed by software flowjo ver 10 (Ashland, OR, USA), LLC. In addition, CTC enumeration by CellSearch™ (Menarini Silicon Biosystems) was performed using mice blood (500 μL) spiked with blood (7 mL) from healthy donors, according to the user‐defined CellSearch™ platform and CellSearch™ Circulating Epithelial Cell Kit [Menarini Silicon Biosystems (Huntingdon Valley, PA, USA); the definition of CTC by Federal Drug Administration (FDA)‐cleared CellSearch™ platform is epithelial cell adhesion molecule (EpCAM)+/cytokeratin (CK)+/DAPI+ but CD45‐ cell]. Fluorescent CTC images along with DAPI were scanned and enumerated by an automated CellBrowser™ software (Menarini Silicon Biosystems), following the manufacturer's guidelines.

### 2.3 Antibodies

For multiparametric flow cytometry, conjugated antibodies FITC‐CD45 (#304054; 1 : 200), FITC‐CD34 (#343504; 1 : 200), FITC‐CD105 (#323204; 1 : 200), FITC‐CD90 (#328108; 1 : 200), FITC‐CD73 (#344016; 1 : 200), FITC HLA‐A/B/C (#311404; 1 : 100), APC‐Cy7‐CD44 (#103028; 1 : 100), BV650‐CD44 (#103049; 1 : 100), and AF647‐Pan‐CK (#628604; 1 : 100) were obtained from BioLegend (San Diego, CA, USA); PE‐Pan‐CK (#5075; 1 : 100) antibody was received from Cell Signaling Technology (Danvers, MA, USA).

For immunohistochemistry, primary antibodies were obtained from the following sources: Anti‐human cocktail of gross cystic disease fluid protein‐15 (GCDFP‐15) + mammaglobin (#906H‐08; 1 : 200) was obtained from Sigma‐Aldrich (St. Louis, MO, USA); CD44 (#960‐MSM2‐P0; 1 : 200) and Pan‐CK (#MSM2‐371‐P0; 1 : 200) antibodies from NeoBiotechnologies (Union City, CA, USA); and Alexa Fluor‐conjugated anti‐mouse, anti‐rabbit secondary IgG antibodies (1 : 500) from Cell Signaling Technology.

### 2.4 CTC‐derived xenografts (CDXs)

The generation of CDXs was performed according to the Institutional Animal Care and Use Committee (IACUC) protocol approved by HMRI. Four‐ to 6‐week‐old immunodeficient mice [NOD.Cg‐Prkdcscid Il2rgtm1Wjl/SzJ (NSG)] were purchased from Jackson Laboratory (Bar Harbor, ME, USA). Flow‐sorted Lin‐neg PBMCs derived from patient blood (8 mL) were injected in anesthetized NSG mice through intracardiac injection under aseptic conditions. To recapitulate the TNBC‐CDX model to the following generation of mice, we used freshly minced metastatic liver tissue from the previous generation of one CDX‐TNBC model (*n* = 1), and incubated it at 37 °C for 1 h with Accumax (Innovative Cell Technologies, San Diego, CA, USA) and DNase I Solution (1 mg/mL) (Stem Cell Technologies, Vancouver, BC, Canada). Dissociated cells were centrifuged (300 ***g*** for 10 min), and the supernatant was collected and strained to obtain single‐cell suspensions (40 μm strainer). We injected 1 × 10E6 liver cells/NSG mouse (*n* = 3 mice per generation) to generate CDX models of subsequent generation through intracardiac injection. Mice were monitored daily until clinical symptoms (hunched, ruffled coat, lethargic posture, etc.) developed for animal euthanization. Mice blood was collected at endpoint through intracardiac puncture with animals being subsequently euthanized. Mice organs were harvested, fixed in 4% paraformaldehyde, and stored in RNAlater (−80 °C) (Thermo Fisher Scientific, Waltham, MA, USA) for downstream analyses. BM cells were harvested by flushing out the femur and tibia with 1× PBS, centrifuged at 300 ***g*** for 10 min, and subjected to PBMC isolation as described above.

### 2.5 Gene expression profiling

RNA was isolated in flow‐sorted CTC/BMRTC populations using NucleoSpin® RNA Isolation Kit (Macherey–Nagel, Bethlehem, PA, USA), according to the manufacturer's protocol. CDX‐derived liver tissue was minced and homogenized in TRIzol™ reagent (Thermo Fisher Scientific). RNA was isolated according to the manufacturer's protocol using RNeasy Mini Kit (Qiagen, Germantown, MD, USA). All RNA samples were immediately provided to the Sequencing and Non‐coding RNA Core (MDACC, Houston, TX, USA) to verify RNA quality by RNA sample integrity (28s/18s ribosomal peaks and their ratio). Whole‐transcriptome amplification and microarray hybridization using the Human Transcriptome Array 2.0 (Affymetrix, Santa Clara, CA, USA) were subsequently performed. Microarray .CEL files were normalized and analyzed by Transcriptome Analysis Console, version 4.0.1 (Affymetrix). Pathway analyses were performed by Ingenuity Pathway Analysis, version 0.7 (Qiagen).

Online patient‐derived GEO microarray datasets were used for primary TNBC tissue (GSE76250, *n* = 31), *de novo* CTCs (GSE99394, *n* = 4) derived from TNBC patients (*n* = 4), and hepatocellular carcinoma (HCC) patients vs normal adjacent tissue (NAT) microarrays (GSE76297, *n* = 57) (Boral *et al*., 2017; Chaisaingmongkol *et al*., 2017; Liu *et al*., 2016). We have chosen only 31 primary triple‐negative breast cancer tissues (pTNBC) microarray paired with normal tissue derived from GSE76250 patient cohort. All microarray datasets were compatible as they were hybridized and analyzed employing the same HTA 2.0 Array Chip (Affymetrix) per manufacturer's guidelines.

### 2.6 Genomic analyses

CTC‐derived xenografts liver tissues were homogenized, and genomic DNA was isolated using QIAamp DNA Mini Kit (Qiagen) according to the manufacturer's protocol. DNA libraries were constructed, and Ion Torrent™ next‐generation sequencing (NGS) was performed for 50 cancer gene panel using Ion AmpliSeq™ Cancer Hotspot Panel v2 (Thermo Fisher Scientific, #4475346) available at the Biostatistics Core of HMRI. Mutational analyses were performed by the Ensembl Variant Effect Predictor and compared with COSMIC mutation database v85 (cancer.sanger.ac.uk) using human hg19 assembly as reference (Forbes *et al*., 2017; McLaren *et al*., 2016). cBioPortal analyses were then carried out to characterize the TNBC‐CTC liver metastasis transcriptomic signature using datasets from 256 TNBC patients (Cerami *et al*., 2012).

### 2.7 Immunofluorescence and immunohistochemistry

FACS‐sorted CTCs/BMRTCs were immunostained with selected primary and secondary antibodies according to a procedure previously described (Vishnoi *et al*., 2015). Briefly, magnified images (100×) were captured using Zeiss Axio Observer microscope Z1 (Carl Zeiss, Jena, Germany) and analyzed by zen2 software (Carl Zeiss).

For immunohistochemistry, harvested and fixed tissues were processed and stained for hematoxylin & eosin (H&E) and respective markers by the Research Pathology Core at HMRI. Images were captured using EVOS XL Cell Imaging System (Thermo Fisher Scientific) (Vishnoi *et al*., 2018).

### 2.8 Survival analyses

We evaluated the survival analyses using the Kaplan–Meier plotter Webtool (http://kmplot.com/analysis/) (Gyorffy *et al*., 2010). Prognostic value of altered transcripts in TNBC patients was assessed by screening for ER, PR, and HER‐2‐negative status in a breast cancer patient cohort (*n* = 3955). Median value of mRNA expression and ‘only JetSet best probe set’ of defined genes were selected for recurrence‐free survival analysis (RFS). Next, female liver cancer patients were selected for overall survival (OS) analyses with *P*‐value < 0.05 considered significant.

## 3 Results

### 3.1 The identification of a transcriptomic signature in *de novo* CTCs isolated from TNBC patients

**Figure 1:**
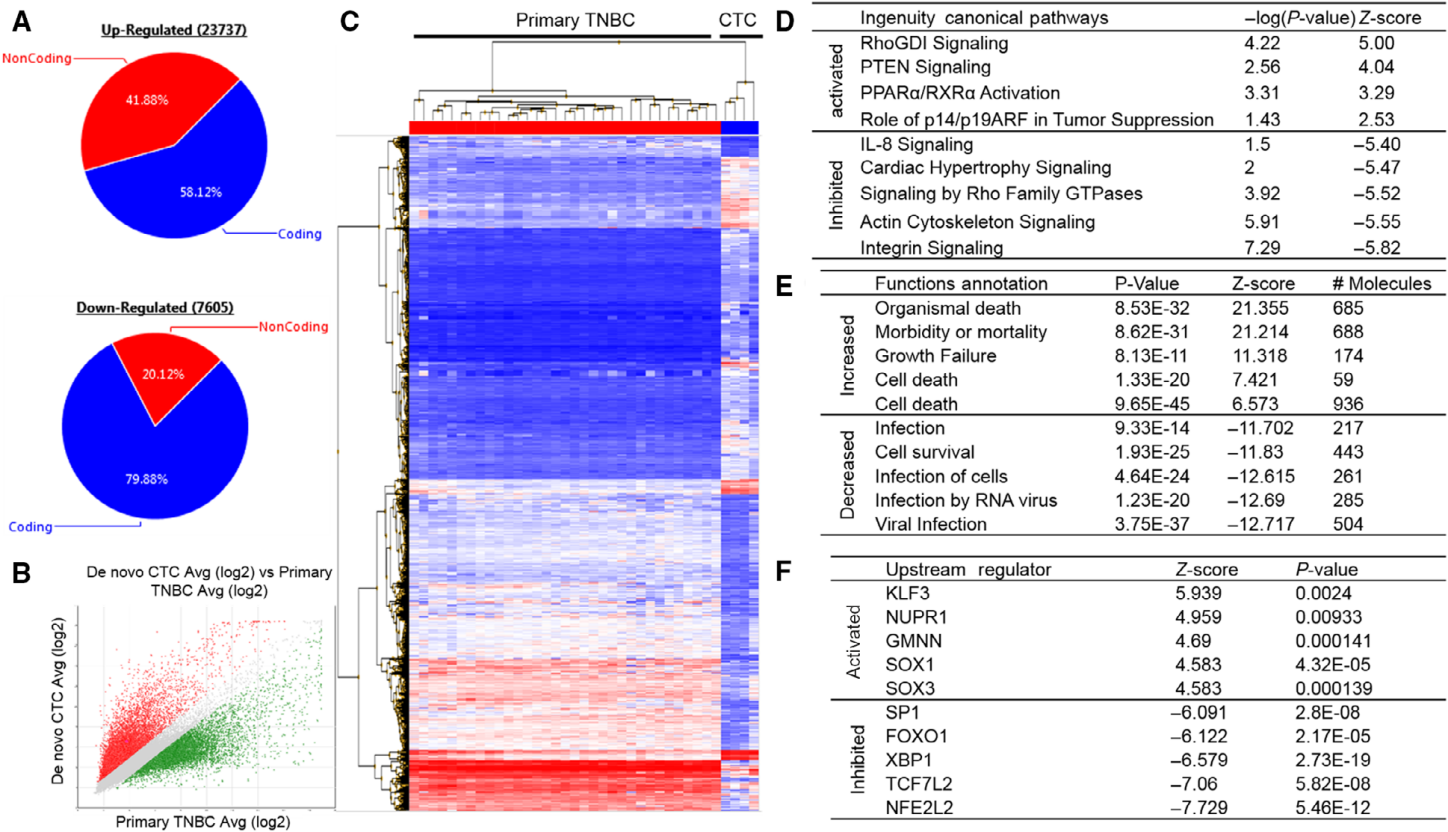
The identification of a *de novo* TNBC‐CTC transcriptomic signature (A) Graph displaying pattern of up‐ and downregulated genes of TNBC patient‐isolated *de novo* CTCs (CD45−/CD34−/CD73−/CD90−/CD105− but CK+/CD44+/CD24− cells) (GSE99394, *n* = 4) vs pTNBCs (GSE76250, *n* = 31); (B) scatter plot showing global gene expression changes in TNBC‐isolated *de novo* CTCs; (C) hierarchical clustering showing distinct transcriptomic signature of TNBC *de novo* CTCs vs pTNBCs (FC = 8; *P*‐value = 005) Two‐way ANOVA test was performed to calculate FC and *P*‐value; (D‐F) table displaying significantly altered top (D) canonical pathways, (E) functional annotation, and (F) upstream transcription regulators of *de novo* CTCs vs pTNBCs.

We have previously reported that patient‐derived CTCs (*de novo* CTCs as isolated from breast cancer patient's PBMCs and immediately injected in NSG mice) contain a distinct transcriptomic signature from primary breast cancer tissues. *de novo* CTCs include heterogeneous subsets whose characteristics relate to dormancy, survival, self‐renewal, invasion, and metastatic competency (Boral *et al*., 2017; Vishnoi *et al*., 2018). Because there is not a definite molecular signature of TNBC‐CTCs nor a universal TNBC‐CTC biomarker available, we selected only *de novo* TNBC‐CTC array from our published database (GSE9934, *n* = 4) and compared with ones from pTNBCs (GSE76250, *n* = 33) (Boral *et al*., 2017; Liu *et al*., 2016). Both databases were obtained employing the same HTA 2.0 array platform and were consistently used throughout this study. Differential gene expression (DGE) analyses revealed 23 737 upregulated (coding = 13 795; non‐coding = 9942) and 7605 downregulated genes (coding = 6075; non‐coding = 1530) [fold change (FC) = 2; *P*‐value = 0.05] (Fig. 1A,B). Unsupervised hierarchical clustering shows the distinct transcriptomic profiling of *de novo* TNBC‐CTCs vs pTNBCs (FC = 8; *P*‐value = 0.05) (Fig. 1C). Ingenuity Pathway Analyses (IPAs) showed the activation of tumor suppressor and nuclear‐ligand receptor signaling pathways and the inhibition of pro‐inflammatory proliferative and invasive signaling pathways (FC = 2; *P*‐value = 0.05) (Fig. 1D). This was also reflected in functional annotation and upstream mechanistic regulator analyses displaying decreased cell survival and prodevelopment functions with the concomitant decrease in cell proliferative and inflammatory functions (Fig. 1E,F) (Boral *et al*., 2017; Vishnoi *et al*., 2018).

### 3.2 Development and validation of a TNBC‐CDX model of liver metastasis

**Figure 2:**
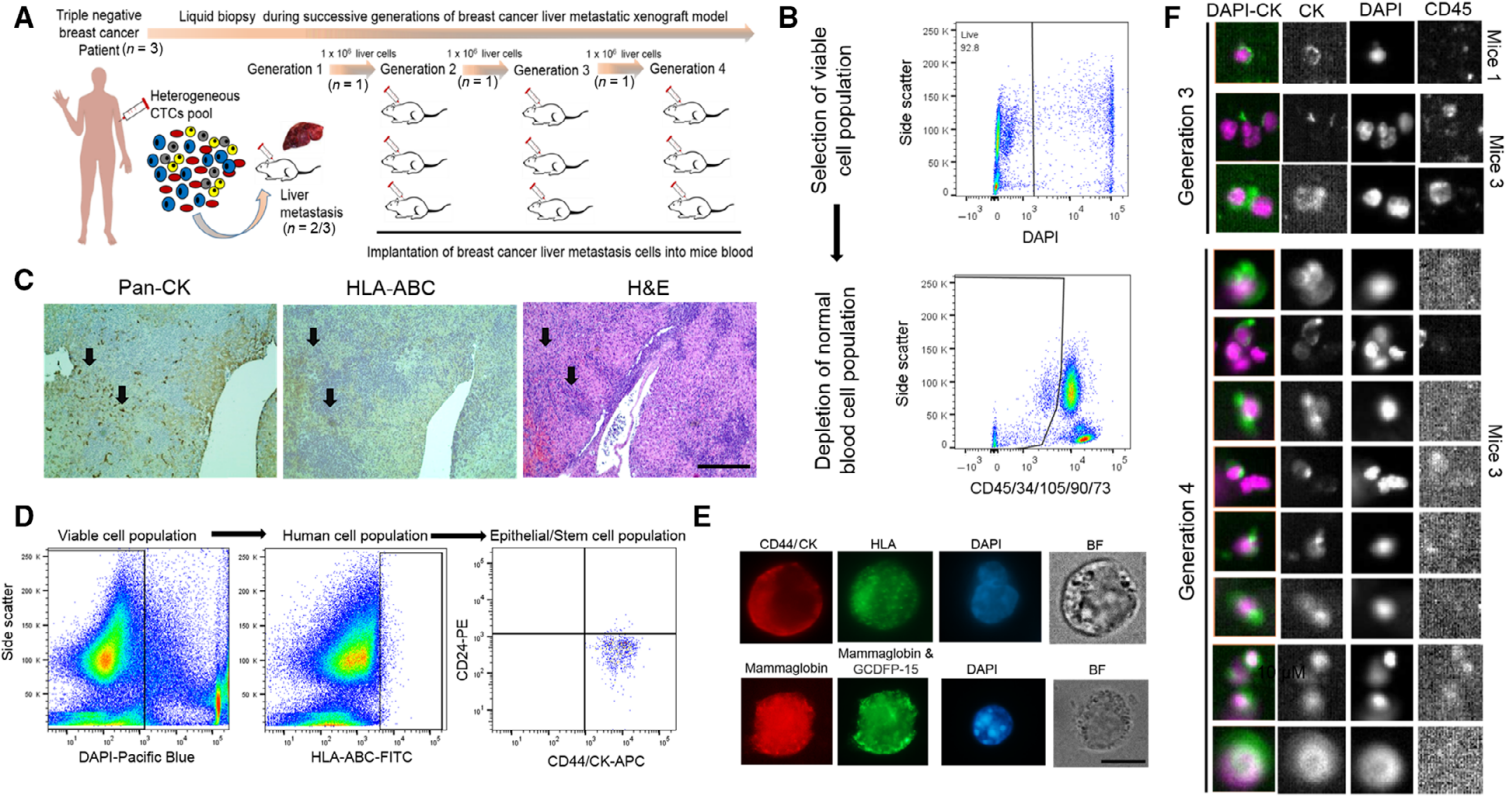
The development and validation of a TNBC liver metastasis CDX model (A) Schematic graph showing the generation of sequential liver metastatic CDXs derived from *de novo* TNBC‐CTCs; (B) multiparametric flow sorting for selection of viable, Lin‐neg/CTC‐enriched cell population (DAPI−/CD45−/CD34−/CD73−/CD90−/CD105− cells) isolated from peripheral blood of TNBC patients; (C) histopathological evaluation of HLA‐ABC, Pan‐CK, and H&E markers staining in the first generation of CDX‐derived liver tissue, scale bar = 50 μm; (D) selection of human breast cancer stem cell population (HLA‐ABC+ and CK+/CD44+/CD24− markers) by multiparametric flow cytometry; (E) representative CTC immunofluorescent images for validation of breast cancer markers (CD44, Pan‐CK, mammaglobin, and GCDFP‐15 markers on flow‐sorted CTC/BMRTC cell populations, scale bar = 10 μm; (F) CellSearch™ enumeration of CTCs (EpCAM+/CK+DAPI+/CD45− cells) from 3rd‐ and 4th‐generation CDXs. Shown are images taken at 10× magnification through CellBrowser™ software (Fig. S1).

We employed a negative depletion strategy to isolate Lin‐neg/CTC‐enriched cell population (CD45−/CD34−/CD105−/CD90−/CD73− cells) from TNBC patients (*n* = 3) through multiparametric flow sorting (Fig. 2A,B and Table S1). Next, to evaluate whether patient‐derived Lin‐neg cells recapitulate tumor progression and metastasis onset, we injected Lin‐neg/CTC‐enriched cell population in immunodeficient mice (NSG) through intracardiac injection. Upon animal euthanization, we harvested blood by intracardiac puncture. Visceral organs (brain, liver, lung, and spleen) and BM were subsequently harvested from these animals (CDX mice). Careful pathological examination of all CDX organs indicated the presence of liver metastasis in 66% of animals injected with Lin‐neg/CTC‐enriched cell population from TNBC patients (Fig. S1). We were able to generate liver metastasis CDX models only from Lin‐neg/CTC‐enriched cell population isolated from the two TNBC patients diagnosed without liver metastasis, however not from the TNBC patient diagnosed with liver mets. First, to prove the human origin of CTC‐induced metastasis, we evaluated tissues for histopathology of human‐specific HLA‐ABC and tumor‐specific Pan‐CK markers staining in harvested organs. We detected specific HLA+/Pan‐CK+ staining in liver detecting extensive macro‐ and micrometastasis (Fig. 2C). Conversely, we did not observe any metastasis in brain, lung, or spleen, which are other common sites of TNBC metastasis in first generation of CDX model. Second, we selectively repropagated TNBC metastatic liver CDX model (*n* = 1) and injected freshly minced CDX‐derived metastatic liver tissue (1.0 × 10E6 cells) intracardiacally in a group of mice and sequentially (four generations of CDX mice, *n* = 3 mice/generation). This strategy helps us to select additional liver metastasis markers, which were present in CTCs that were not colonized yet. We recapitulated TNBC liver metastasis CDX model in these generations but did not observe any difference in OS (> 2 months) (Fig. 2A). Third, to validate this model, we performed immunohistochemistry in serial liver sections for staining of HLA‐ABC/Pan‐CK in addition to mammary cell‐specific GCDFP‐15 and mammaglobin markers (Fig. 2C). HLA+, Pan‐CK+, and GCDFP‐15 + mammaglobin‐positive areas confirmed the presence of human breast cancer cells in the fourth generation of TNBC‐CDX model of liver metastasis (Fig. S1a,b). Fourth, to confirm the neoplastic identity of these cells, we carried out extensive genomic mutational analyses of TNBC liver metastatic tissue derived from each generation of CDX mice (Ion Torrent AmpliSeq 50 cancer genes panel). We analyzed a total of 111 mutations, finding cosmic mutations in NRAS, PTEN, KRAS, IDH2, STK11, ERBB4, VHL, PIK3CA, APC, SMO, and NOTCH1 genes uniquely present in all sequential generations of CDX‐derived liver tissue (Table 1 and Fig. S2); of note, PIK3CA gene cosmic mutation (COSM21451) is known to be associated with HCC (Forbes *et al*., 2017). Lastly, we performed flow‐sorting analyses to isolate HLA+/CD44+/Pan‐CK+ cell populations from blood and BM, and they were validated by further immunofluorescent staining of human HLA, CD44, Pan‐CK, and GCDFP‐15+ mammaglobin markers for the presence of human‐specific mammalian tumor stem cell phenotype (Fig. 2E). CellSearch™ CTC interrogation (CellSearch™ is the only FDA‐cleared platform for clinical CTC interrogation (Joosse *et al*., 2015)) in CDX‐derived blood (500 μL) from 3rd and 4th generation of mice was performed. We observed increased numbers of EpCAM+/Pan‐CK+/DAPI+ but CD45‐ cells (CellSearch™ CTCs) in 4th‐generation vs 3rd‐generation CDX mice (Fig. 2F). Thus, the TNBC‐CDX model recapitulates liver metastasis in sequential CDX generations by displaying similar genomic profiling and disseminated tumor cells (CTCs and BMRTCs).

**Table 1:** Cosmic mutations consistently detected in all generations of TNBC‐CTC liver metastasis CDX models. HCC, hepatocellular carcinoma.

Gene	COSMIC ID	cDNA change	Protein change	Predisposing disease
NRAS	COSM1162157	c87A>G	pV29V	Malignant melanoma
NRAS	COSM1162156	c60A>G	pT20T	Malignant melanoma
PTEN	COSM921141	c870delA	pE291fs*16	Carcinoma
KRAS	COSM2202566	c126G>A	pK42K	Squamous cell carcinoma
KRAS	COSM2202568	c114T>C	pD38D	Squamous cell carcinoma
IDH2	COSM3355752	c429G>C	pL143L	Glioma
STK11	COSM3357078,COSM3357079	c102C>T	pV34V	Lymphoid neoplasm
ERBB4	COSM5672204,COSM5672205	c2799A>T	pL933F	Small cell carcinoma
ERBB4	COSM1165236	c2778G>A	pT926T	Adenoma
VHL	COSM17955	c285G>A	pP95P	Carcinoma
PIK3CA	COSM21451	c3075C>T	pT1025T	HCC, carcinoma
PIK3CA	COSM5418401	c3087T>G	pD1029E	Carcinoma
PIK3CA	COSM28545	c3123A>T	pK1041N	Carcinoma
APC	COSM18869	c4308delT (Deletion)	pS1436fs*37	Carcinoma
APC	COSM296347	c4314delA	pP1439fs*34	Carcinoma, adenoma
APC	COSM19702	c4323delA	pP1442fs*31	Adenoma
APC	COSM26700	c4464A>T	pL1488F	Carcinoma
APC	COSM29572	c4494delT	pD1498fs*9	Carcinoma
APC	COSM5369891	c4497_4497delA	pS1501fs*6	Carcinoma
SMO	COSM5351376	c1539G>A	pP513P	Carcinoma
SMO	COSM1085436	c1623G>A	pT541T	Endometrioid and ductal carcinoma
NOTCH1	COSM305943	c4793G>C	pR1598P	Lymphoid neoplasm
NOTCH1	COSM4496519	c4776C>T	pF1592F	Carcinoma
NOTCH1	COSM4774957	c4738A>C	pM1580L	Lymphoid neoplasm

### 3.3 The identification of a TNBC metastasis gene signature in CDX‐derived cell populations

**Figure 3:**
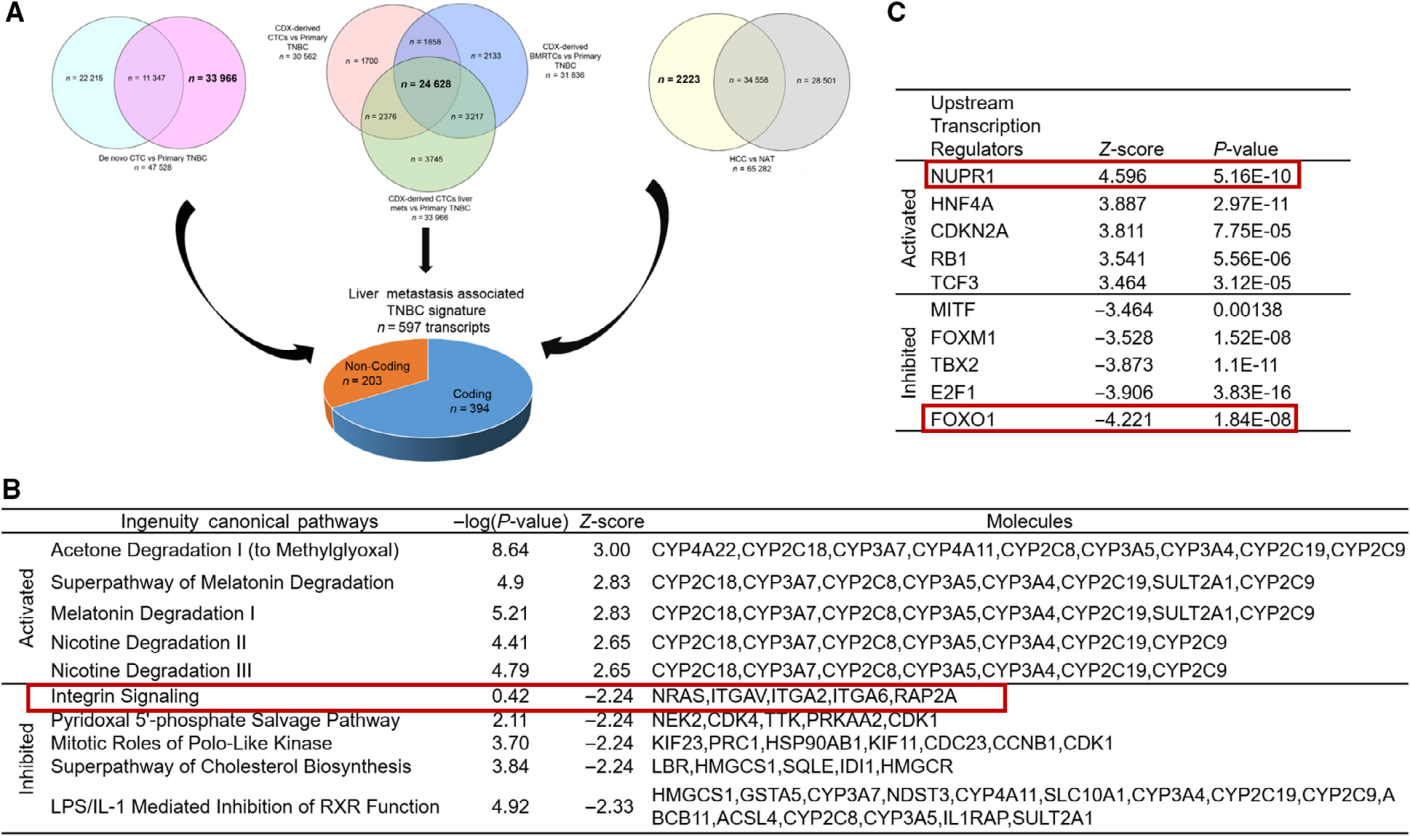
Transcriptomic analyses of the TNBC liver metastasis signature (A) Venn diagram identifying TNBC‐CTC liver metastasis signature. Venn diagram represents DGE of *de novo* CTCs vs pTNBCs, CDX‐derived cell populations (CTCs, BMRTCs, and liver metastasis tissue obtained from the same animal) vs pTNBCs, and HCCs vs NAT. Overlapping genes (shown in bold) were used to identify a signature of liver metastasis in TNBC patients; (B and C) top five activated and inhibited (B) canonical pathways and (C) upstream transcription regulators associated with TNBC liver metastasis. Red box represents common transcriptomic signatures present in TNBC patient‐derived *de novo* CTCs and liver metastasis.

As first step, we isolated RNA from flow‐sorted HLA+/CK+/CD44+/CD24‐ cell population from paired blood (*n* = 4), BM (*n* = 4), and liver tissue (*n* = 4) derived from each generation of CDX mice (i.e., CTC/BMRTC populations). Next, we performed whole‐transcriptome profiling of CDX‐CTCs, BMRTCs, and liver tissue obtained from the same animal and using the same microarray platform HTA 2.0 (Affymetrix™) and protocol. We compared transcriptomics of all CDX‐derived populations (CTCs, BMRTCs, and liver tissue) vs pTNBCs, and analyzed 24 628 significantly overlapping genes (FC = 2; *P*‐value = 0.05). This represented a first‐level decipheration of TNBC‐CDX‐derived population, which was found to be distinct from pTNBC transcriptomics (Fig. 3A). Additionally, 1700 transcripts in CDX‐derived CTC population, 2133 transcripts in CDX‐derived BMRTCs, and 3745 transcripts in CDX‐derived liver tissue were uniquely expressed in the respective CDX‐derived cell population vs pTNBCs. Second, we compared the CDX‐derived overlapping transcripts (*n* = 24 628) with previously analyzed *de novo* TNBC‐CTC transcriptomics (*n* = 33 966). We discovered 19 892 significantly altered transcripts present in *de novo* CTCs along with all CDX‐derived cell population vs pTNBCs (common gene signature of TNBC‐CTCs). Furthermore, we compared transcripts of *de novo* and CDX‐derived CTC cell populations, and detected 22 801 overlapping transcripts, 8541 and 7761 transcriptomic signatures specific of respective *de novo* and CDX‐derived CTC population vs pTNBCs. Third, we performed pancancer analysis to identify commonalities and differences in key biological processes that are dysregulated in cancer cells from diverse lineages. This strategy identified biomarkers and signaling pathways to define mechanism unique for homing and colonization of tumor cells in the liver‐specific niche. Gene expression array databases of TNBC‐derived liver metastasis using same array platform are not available; therefore, we employed a signature of HCC database (GSE76297, *n* = 57) to discover a gene signature implicated in liver metastasis of our TNBC‐CDX model (Chaisaingmongkol *et al*., 2017). DGE of HCC vs paired NAT shows 2223 significantly altered transcripts (552 upregulated and 671 downregulated transcripts; FC = 2.0; *P*‐value = 0.05), representing a gene signature of liver tumor cells responsible for homing and colonization at an organ‐specific niche. To identify a TNBC liver metastasis signature, we then compared these significantly altered transcripts with a TNBC signature analyzed above (*n* = 19 892 transcripts) in *de novo* CTCs and CDX‐derived cell populations (CTCs, BMRTCs, and metastatic liver tissue obtained from the same animal) vs pTNBCs. We observed 597 common gene transcripts (coding 394 and non‐coding 203 genes) representing a TNBC liver metastasis gene signature (Fig. 3A). IPAs show activation of xenobiotic pathways reflecting the normal metabolic function of liver present in *de novo* CTCs as well as in CDX‐derived cell populations (Fig. 3B). The predicted activation of upstream transcription regulators NUPR1, HNF4A, CDKN2A, RB1, and TCF3, and the corresponding inactivation of MITF, FOXM1, TBX2, E2F1, and FOXO1 transcription regulators were detected (Fig. 3C). Three microRNAs—MIR4450, MIR4466, and MIR4737—were significantly upregulated in all analyzed populations (*de novo* CTCs and CDX‐derived cell populations) vs pTNBCs. Further, we analyzed predicted targets of all three miRNAs using Webtools: miRBase (http://www.mirbase.org/) and TargetScanVert (http://www.targetscan.org). We identified three common targets in miRBase and TargetScanVert of MIR4450: MCM6, SLC17A2, and TUBB genes that were downregulated in the identified TNBC liver metastasis CTC signature. Downregulation of these genes delays cell growth and induces the senescent phenotype (Chang *et al*., 2010; Liu *et al*., 2018b). Conversely, we did not detect any predicted targets of MIR4466 and MIR4737 in TNBC liver metastasis signature (597 genes). Notably, MIR4450 gene (Accession #MI0016795) was the top upregulated transcript in *de novo* CTCs and CDX‐derived BMRTC population (FC = 34.29; *P*‐value = 8.84E‐17). This suggests that MIR4450 may play a role in dormancy‐induced CTC phenotype, balancing self‐renewal and cell survival properties.

### 3.4 Survival analyses

**Figure 4:**
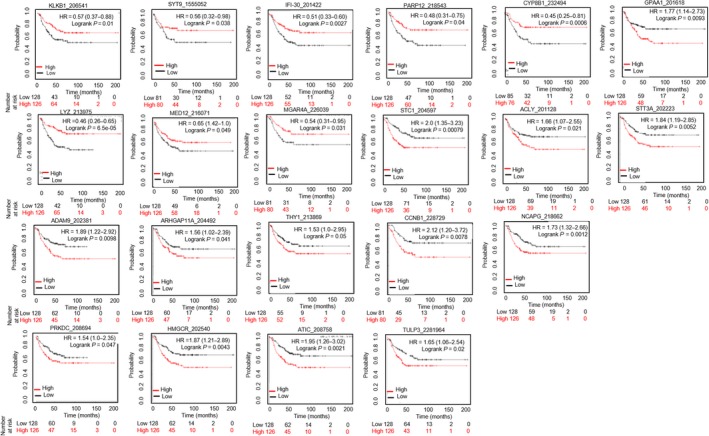
Survival analyses of the TNBC liver metastasis signature. RFS analyses were performed in TNBC patients (*n* = 256) (KM plot.com). Gene, their selected probe ID, hazard ratio (HR), and log‐rank *P*‐value are indicated at the top of each survival graph. Red color indicates high expression, while black color indicates low expression of respective gene. *X*‐axis represents patient number and survival (in months), while the *Y*‐axis represents probability.

To evaluate the association of relapse‐free survival (RFS) in TNBC patients, we extended KM plot survival analyses in 597 transcripts of the identified TNBC liver metastasis gene signature using TNBC patient database (Fig. 4). Combined gene expression and survival data analyses show that upregulation of KLKB1 (HR = 0.57), SYT9 (HR = 0.56), and CYP8B1 (HR = 0.45), and downregulation of IFI30 (HR = 0.51), PARP12 (HR = 0.48), LYZ (HR = 0.42), MED12 (HR = 0.65), and MGAT4A (HR = 0.54) were significantly associated with favorable RFS of TNBC patients (*P*‐value < 0.05). Conversely, downregulation of ACLY (HR = 1.66), ADAM9 (HR = 1.89), ARHGAP11A (HR = 1.56), ATIC (HR = 1.95), CCNB1 (HR = 2.12), GPAA1 (HR = 1.77), HMGCR (HR = 1.87), NCAPG (HR = 1.73), PRKDC (HR = 1.54), STC1 (HR = 2.09), STT3A (HR = 1.84), THY1 (HR = 1.53), TULP3 (HR = 1.65), and ZNF704 (HR = 1.92) was associated with poor RFS in TNBC patients (*P*‐value < 0.05, Fig. 4). Further, we assessed survival genes associated with OS of liver cancer female patients using KM plot (Fig. S3). We found COLEC10, GPAA1, CD5L, NCSTN, SNX27, GOLPH3L, and HIST2H2AC genes were associated with worst OS (HR < 1) in female liver cancer patients. LAPTM4B, RIPK2, COPA, NUF2, CDD1L, CHML, EXO1, and FOXM1 were associated with favorable outcome of liver cancer female patients (HR > 1).

### 3.5 Genomic characterization of the TNBC liver metastasis signature

**Figure 5:**
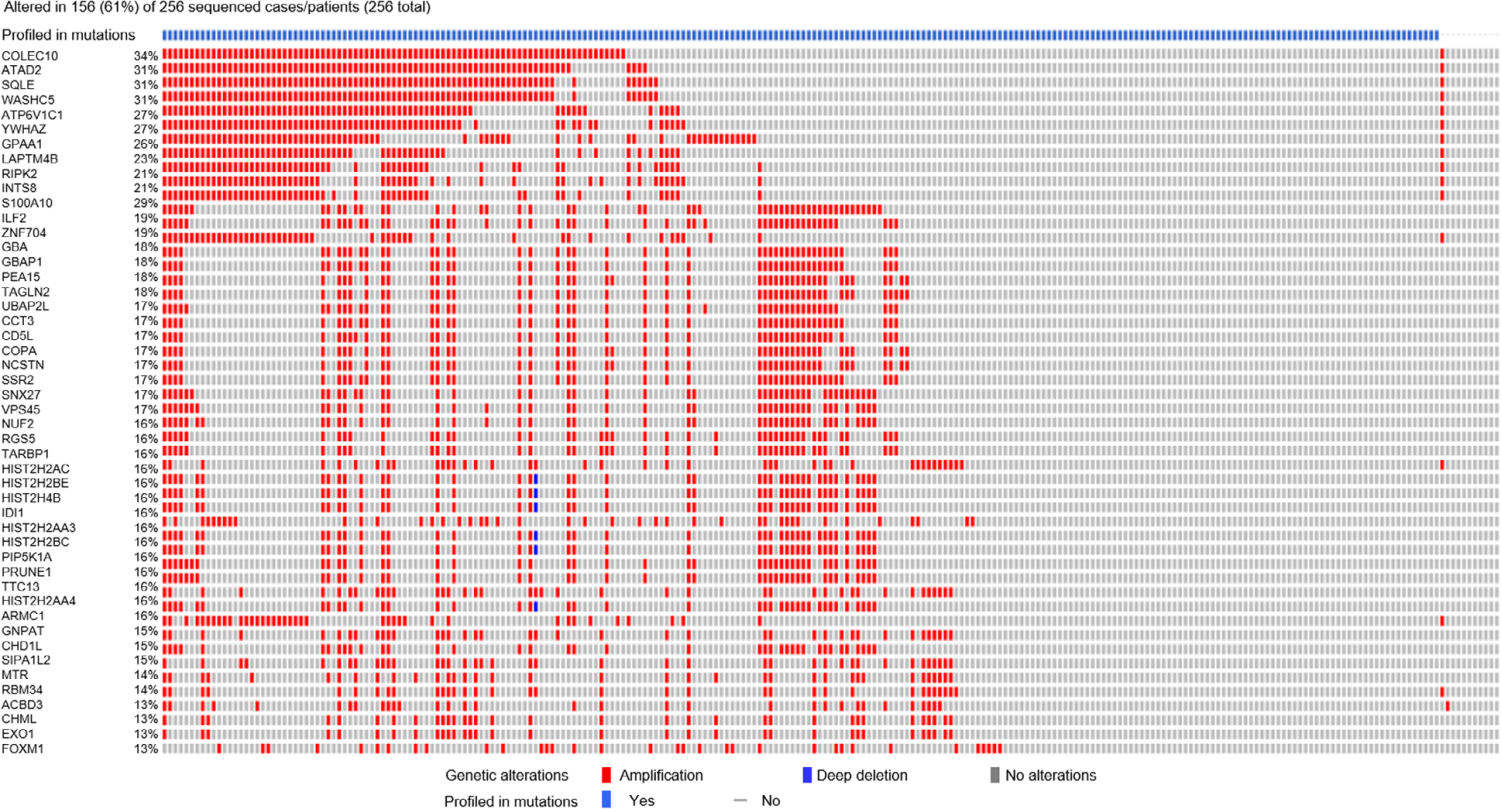
Top 50 altered genes associated with the TNBC liver metastasis signature. cBioPortal analysis was performed in TNBC patients (*n* = 256) to display the top 50 altered genes associated with the TNBC liver metastasis signature. Red and blue colors indicate amplification or deletion of respective genes.

**Figure 6:**
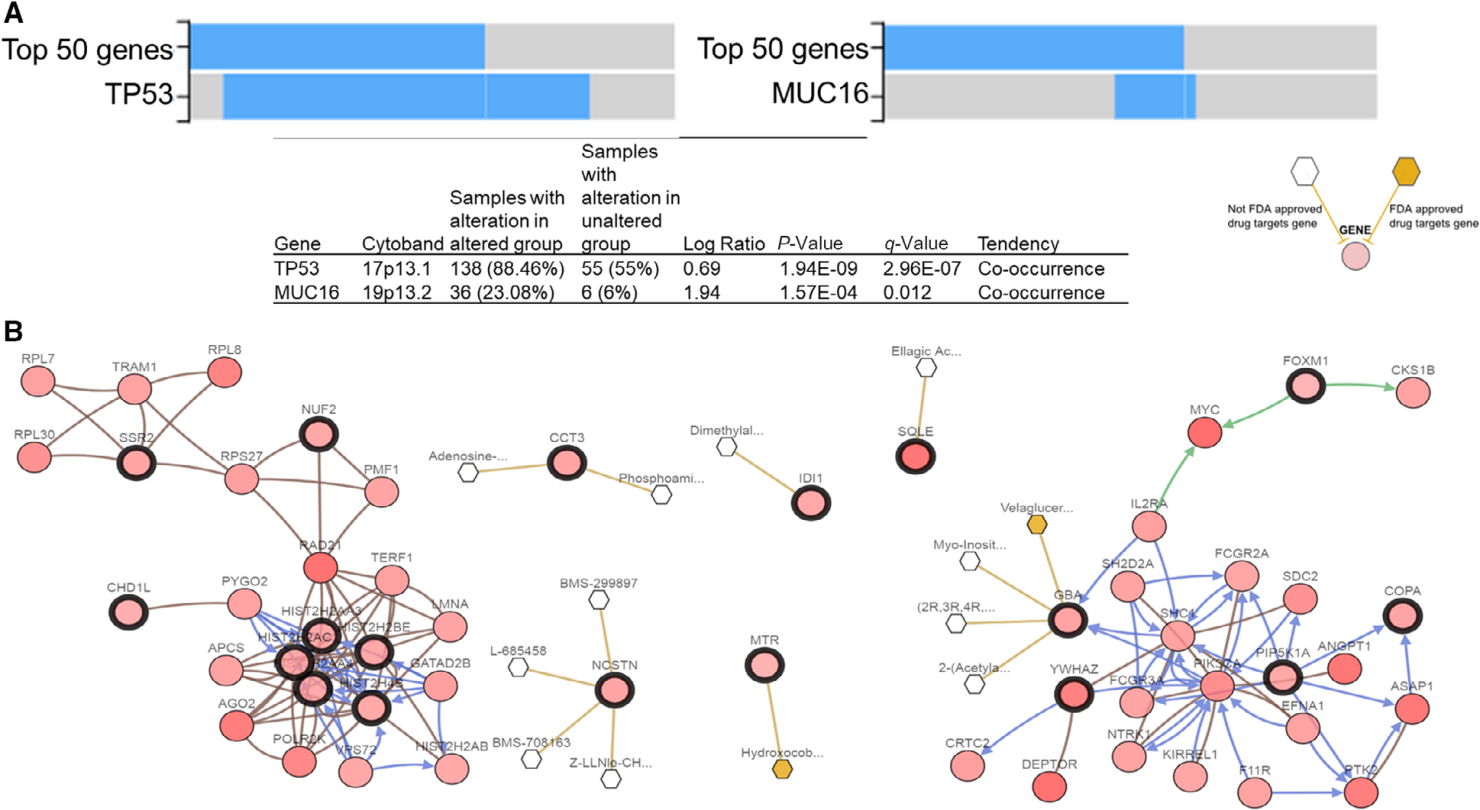
Genomic characterization of the TNBC liver metastasis signature cBioPortal analysis of TNBC patients (*n* = 256) showing (A) enrichment analysis for co‐occurrence of top 50 altered genes with TP53 and MUC16 (upper panel) and their respective values (bottom panel); (B) network analysis of top 50 altered genes showing interactions with 16 hub genes (dark circles) as potential drug candidates. White/yellow hexagons represent FDA‐approved/not approved cancer drugs against the target gene, respectively.

We performed cBioPortal genomic analyses of TNBC liver metastasis signature. We observed 193 (67%) genomic alterations across 256 TNBC patients. Figure 5 shows genomic alteration of top 50 genes (> 13%) present in 256 TNBC patients. Pairwise co‐occurrence analyses of the 50 genes displayed 502 gene pairs to be significantly associated with TNBC patients, of which 423 gene pairs showed high risk (HR > 3; *P*‐value < 0.001, Table S2). We also identified homozygous copy number deletion of histone genes (HIST2H2AC, HIST2H2BE, HIST2H4B, HIST2H2AA3, HIST2H2BC, and HIST2H2AA4) in one patient from TCGA TNBC cohort. Further, analyses of gene enrichment indicated that genomic alterations in these genes were associated with co‐occurrence of TP53 (log ratio 0.68; *P*‐value = 1.94E‐09) and MUC16 mutations (log ratio 1.94; *P*‐value = 1.57E‐04) (Fig. 6A). Network analyses displayed 16 hub genes (SQLE, CCT3, NCSTN, GBA, SSR2, YWHAZ, MTR, CHD1L, NUF2, SSR2, PIP5K1A, COPA, IDI1, HIST2H2AC, HIST2AA3, and HIST2H4B) and their interactions with other genes (Fig. 6B). We also identified six predicted SQLE, CCT3, IDI1, GBA, MTR, and NCSTN cancer drug target genes. Further, we validated high expression of NCSTN, NUF2, YWHAZ, and PIP5K1A genes to be associated with liver metastasis in *de novo* TNBC‐derived Lin‐neg/CTC‐enriched cell population.

## 4 Discussion

The concept of liquid biopsy relates to the development of procedures monitoring the evolution of cancer in the patient and the emergence of therapy resistance and recurrence (minimal residual disease). Systematic analyses of CTCs and their interrogation can provide relevant information directly in real time and non‐invasively to identify candidate biomarkers clinically relevant for diagnosis, prevention, and therapy (precision oncology). CDX models can provide better tool to study specific organ‐competent CTC signature over time by the sequential generation of CDX models. However, these CDX models can be less relevant for individualized patient therapy, but provide critical information to evaluate the metastasis‐competing state of clinical CTCs (Alix‐Panabieres and Pantel, 2014). Here, we provide first‐time evidence of molecular profiling of *de novo* TNBC‐CTCs (Lin‐neg but CK+/CD44+/CD24‐ cell population), along with novel insights of genes associated with mechanism of CTC survival; notably, identifying a gene signature associated with CTC metastatic potency to liver (Fig. 1A–F). We report the successful establishment of TNBC liver metastasis CDX models and their comprehensive genomic and immune phenotyping characterization to recapitulate the biology of TNBC liver metastasis at each CDX generation by injecting Lin‐neg/CTC‐enriched cell populations directly isolated from peripheral blood of TNBC patients (Fig. 2A) and by assessing the human origin of breast cancer cell populations in circulation as well as at the metastatic liver site (Figs 2A–E, S1 and S2, and Table 1). CTC analyses by CellSearch™ (a FDA‐cleared platform for clinical testing of EpCAM+ CTCs) of these CDX models demonstrated an increased number of CTCs and tumor burden in the later generations of CDX mice (Fig. 2F). Second, to understand molecular landscapes of CTCs in our TNBC‐CDX model, we analyzed gene expression profiling of various CDX‐derived populations according to the liver metastasis onset. We identified 24 628 overlapping transcripts (Fig. 3A); of these, 19 892 transcripts shared gene expression profiling of *de novo* TNBC‐CTCs. These findings suggest that the TNBC‐CTC liver metastasis CDX model we have established has abilities to discriminate the metastatic potency of CTCs during disease progression *in vivo*.

Liver metastasis is a preeminent cause of breast cancer patients’ death (Wyld *et al*., 2003). However, development of organ‐specific metastasis is not random, rather a selective and specific process (Joosse *et al*., 2015; Muller *et al*., 2010; Riethdorf *et al*., 2008). The genomic characterization of CTCs confirms notions that these cells are highly heterogeneous with some subsets capable to survive as disseminated cells for prolonged period of time: Only few clones can interact with a specific target organ microenvironment or fostering CTC colonization at the organ site by the formation of a metastatic niche (Alix‐Panabieres *et al*., 2008; Joosse *et al*., 2015; Muller *et al*., 2009; Riethdorf and Pantel, 2008). Dissecting CTC molecular properties and mechanisms can thus promote the use of CTC tests (liquid biopsy) as clinically useful tools to predict the risk of metastatic recurrence at a specific organ and to potentially drive therapy. As proof of concept, we focused on comparing gene expression databases of HCC, CDX‐derived populations, and *de novo* CTCs to evaluate similar molecular signature between TNBC and HCC cross‐cancer types. We identified a transcriptomic signature (597 genes) associated with clinical TNBC liver metastasis (Fig. 3A). Further analysis revealed 22 survival genes that may predict the risk for liver metastasis in TNBC patients. This can be considered as first step to develop a liquid biopsy prognostic test applicable to clinical settings (Fig. 4). However, mechanistic studies will be required to prove relevance of these 22 genes relative to CTC survival and their biology associated with liver metastasis‐free progression in TNBC patients. For example, comparative pathway analyses show that transcription regulator Nuclear Protein1 (NUPR1) is consistently activated in *de novo* TNBC‐CTCs and CDX‐derived cell populations (Figs 1F and 3C). NUPR1 binds with Tp53 and promotes cell growth and survival of chemotherapeutic‐resistant breast cancer cells (Clark *et al*., 2008). An autoregulatory loop of NUPR1/RELB/IER3/RUNX2 pathway plays a key role in disease progression of HCC by regulating cell growth, migration, invasion, and chemoresistance (Emma *et al*., 2016). Further investigations of the mechanisms of NUPR1/Tp53 and therapeutic targeting will be critical for progression of liver metastasis in TNBC patients. Comprehensive multiparametric gene expression profiling and TCGA analyses of 256 TNBC patients identified top molecular altered genes and their association with TNBC progression (Fig. 5). Identifying their relative mechanism of co‐occurrence with Tp53 and MUC16 mutations in pathogenesis of TNBC liver metastasis can be helpful in determining drug relapse immunotherapy (Fig. 6A).

We also identified six drug candidate target genes (SQLE, CCT3, IDI1, GBA, MTR, and NCSTN), which can be used as prerequisite to develop novel therapeutic interventions (Fig. 6B). For example, the drug candidate nicastrin (NCSTN) is a ƴ‐secretase clinically relevant in breast invasive carcinoma and associated with worst OS of liver carcinoma (Filipovic *et al*., 2014; Woo *et al*., 2009). Its pharmacological inhibition abolished various cell functions such as cell proliferation, invasion, extracellular matrix degradation, invadopodia extension, and trans‐endothelial extravasation, via Notch and mTOR signaling pathway regulation (Filipovic *et al*., 2014; Woo *et al*., 2009). NCSTN also regulates ƴ‐secretase‐independent cell death via phosphoinositide 3‐kinase/Akt and Tp53‐dependent pathways (Pardossi‐Piquard *et al*., 2009). Digital analysis of RNA from breast cancer CTC was recently reported, interrogating treatment responses of both localized and metastatic breast cancers (Kwan *et al*., 2018). However, CTCs were not interrogated for their signatures and validation in sequential generations of CDX models as reported here.

To our knowledge, this is the first study to provide a CTC‐associated TNBC liver metastasis signature. A limitation of this study is the unavailability of matched TNBC primary and liver metastasis datasets required to perform comprehensive gene expression analysis for classifying the TNBC liver metastasis CTC signature. Further, although we performed gene expression analysis of CDX‐derived cell populations in sequential generations of mice, we cannot exclude sampling bias due to the small sample size. Investigations using larger sample cohorts will be needed to generate additional CDX models and determine the mechanistic, therapeutic, and prognostic relevance of our identified TNBC‐CTC liver metastasis gene signature. However, the CDX‐CTC approach reported here can be critically relevant to discover CTC signatures specific to other organ sites beyond liver, recapitulating disease progression in the patient.

## 5 Conclusions

We developed and employed CDXs as improved models to reflect TNBC progression *in vivo*. We identified a 597 gene signature associated with TNBC liver metastatic patients. Liquid biopsy‐based testing of identified 22 survival genes can be used as prognostic marker for liver metastasis in TNBC patients. Further, drug candidate biomarkers may foster the use of precise therapeutic approaches to eliminate residual cells in metastatic TNBC patients. Future mechanistic investigations and prospective studies are needed to delineate the role of these genes in TNBC liver metastasis CDX models.

## Conflict of interest

The authors declare no conflict of interest.

## Author contributions

MV designed the experiments and interpreted the results. MV, HNL, YW, and DB performed the experiments. MV and YW analyzed the data. ATS coordinated provision of samples to the laboratory. DSH provided clinical patient blood samples and clinical input. MV and DM wrote the manuscript. DM supervised the study. All authors read and approved the final manuscript.

## Supporting information


**Fig. S1.** Histopathological images showing liver metastasis in sequential CDX models.
**Fig. S2.** Identical genomic mutational pattern of CDX model (1‐4 generations) derived from sequencing of 50 cancer genes through AmpliSeq Ion Torrent Sequencing panel.
**Fig. S3.** Survival genes of top 50 genomic altered genes derived from cBioPortal analysis.
**Table S1.** Clinical parameters of TNBC patients used in this study.
**Table S2.** Twenty‐four gene pairs with significant 502 co‐occurrent alterations.
