# Supplementary material for: The identification of a TNBC liver metastasis gene signature by sequential CTC‐xenograft modeling
Source: Mol Oncol. 2019 Jun 19;13(9):1913–26. doi: 10.1002/1878-0261.12533 (PMC6717757; doi:10.1002/1878-0261.12533)
Supplement: Supplementary file 1 — Fig. S1. Histopathological images showing liver metastasis in sequential CDX models. Fig. S2. Identical genomic mutational pattern of CDX model (1‐4 generations) derived from sequencing of 50 cancer genes through AmpliSeq Ion Torrent Sequencing panel. Fig. S3. Survival genes of top 50 genomic altered genes derived from cBioPortal analysis. Table S1. Clinical parameters of TNBC patients used in this study. Table S2. Twenty‐four gene pairs with significant 502 co‐occurrent alterations. [file MOL2-13-1913-s001.pdf]

The identification of a TNBC liver metastasis gene signature by sequential CTC-xenograft modelling

Monika Vishnoi[1], Haowen Liu[1], Wei Yin[1], Debasish Boral[1], Antonio T. Scamardo[2], David Hong[2], and Dario Marchetti[1*]

[1]Biomarker Research Program Center, Houston Methodist Research Institute, Houston, TX.

[2]Department of Investigational Cancer Therapeutics, The University of Texas MD Anderson Cancer Center, Houston, 77030 TX, USA.

[*]Correspondence and requests for materials should be addressed to Dario Marchetti. 6670 Bertner Avenue, Houston, TX. Phone: 713-363-7769; Fax: 713-363-7717; E-Mail: dmarchetti@houstonmethodist.org

Supplementary Table S1: Clinical parameters of TNBC patients used in this study

Patient ID	Primary cancer	Metastatic site	Her-2	ER/PR	PIK3CA & BRCA1/2 mutation	Stage at diagnosis	Ki67 Results
Pt 1	Breast	Lung and node	Neg	Neg/ Neg	Wild type	T2N3b	Positive
Pt 2	Breast	Bones, nodes, lung and liver	Neg	Neg/Neg	Wild type	4	Positive
Pt 3	Breast	Node, lung and bone	Neg	Neg/Neg	Wild type	4	Not available

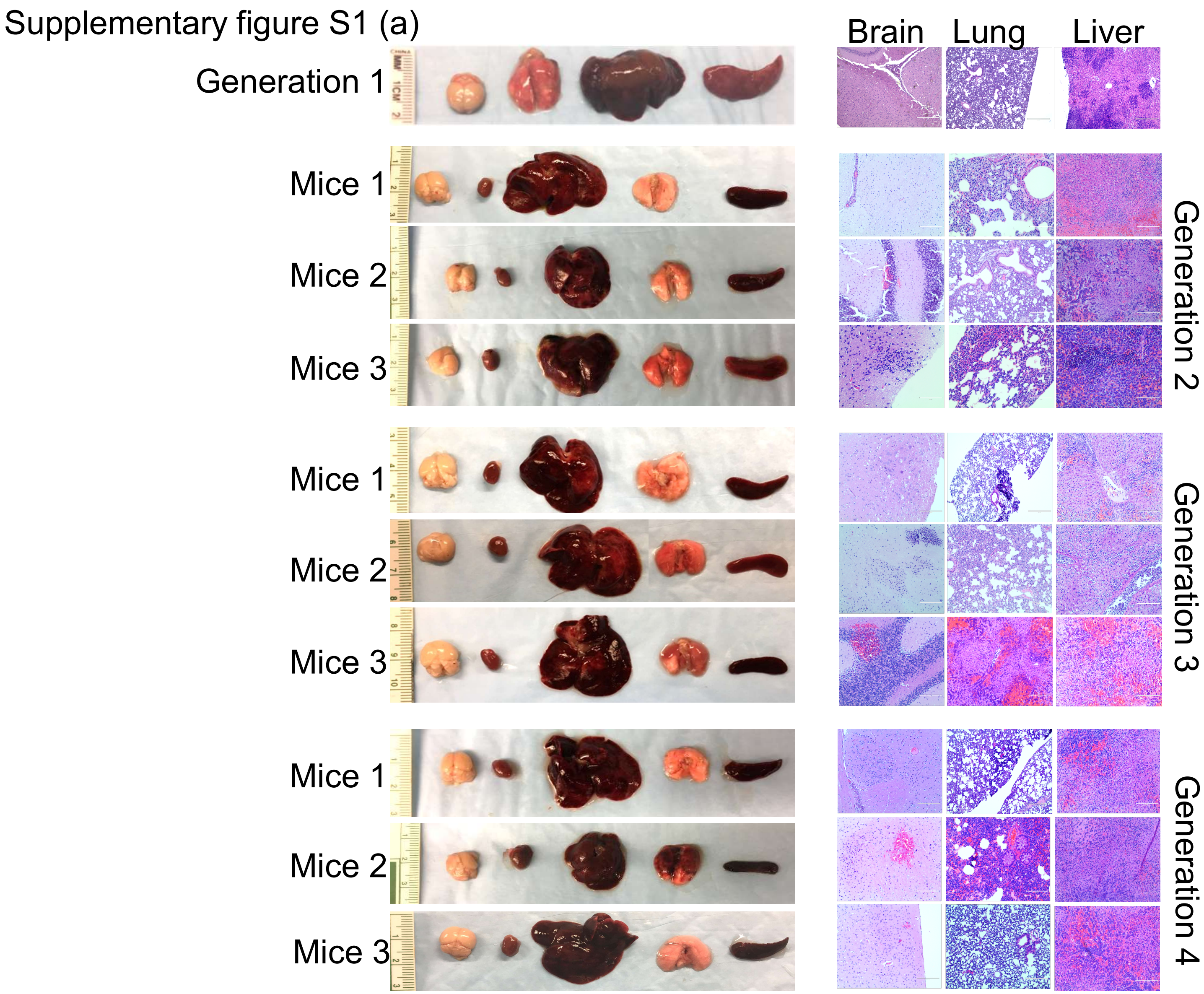

Supplementary figure S1a: Left panel showing macroscopic liver metastasis vs other organs (brain, heart, lung and spleen) and right panel showing H&E staining of brain, lung and liver derived from liver metastatic TNBC CDX mouse for each successive generations.

Supplementary figure S1 (b)

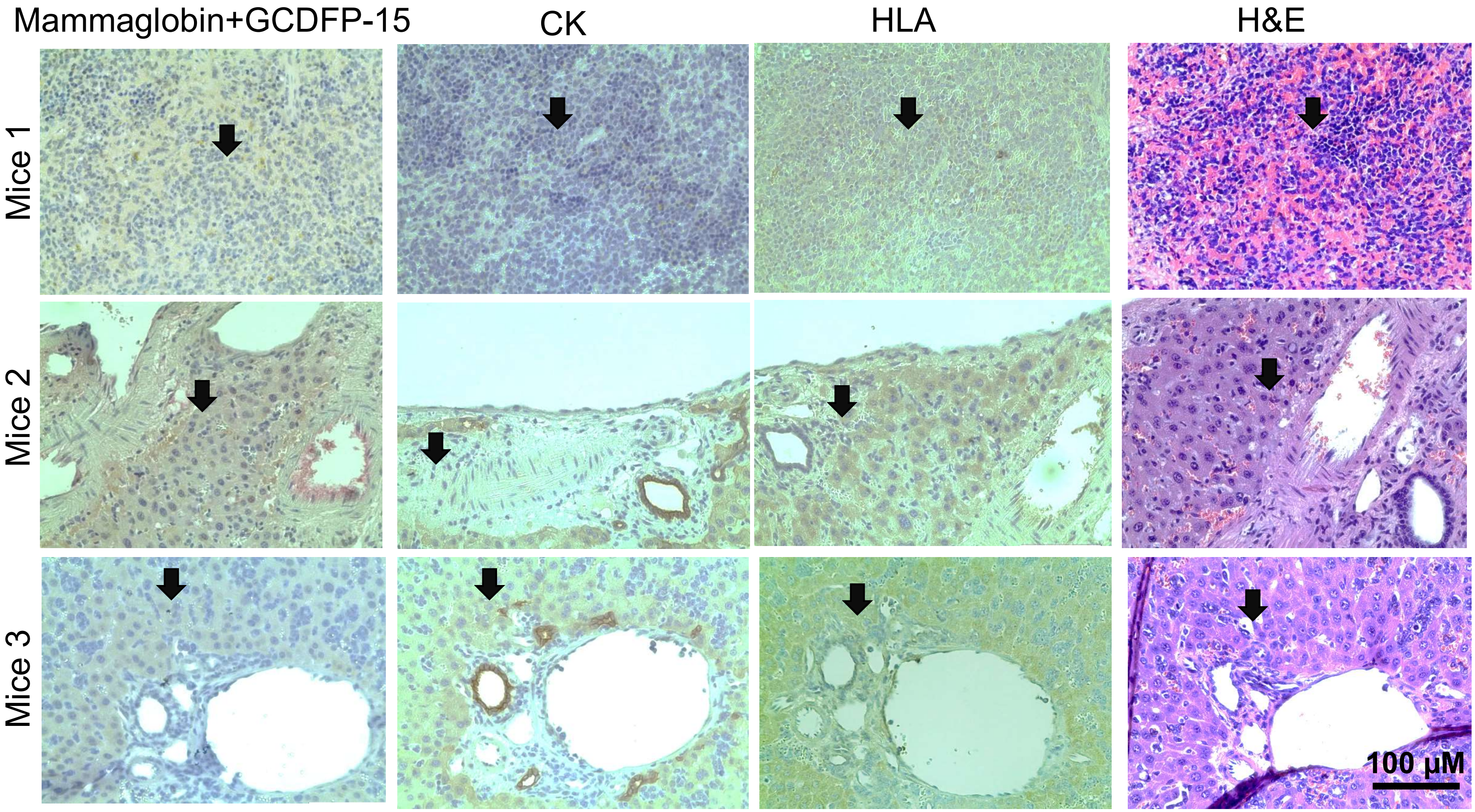

(b) Histopathological evaluation showing staining of human (HLA-ABC), epithelial tumor (Pan-CK) and breast origin (mammoglobin+GCDFP15) markers in CDX-derived liver tissue of fourth generation. Left panel showing anti-GCDFP-15+ anti-mammaglobin (brown color) and anti-GCDFP-15 only (red color) markers staining

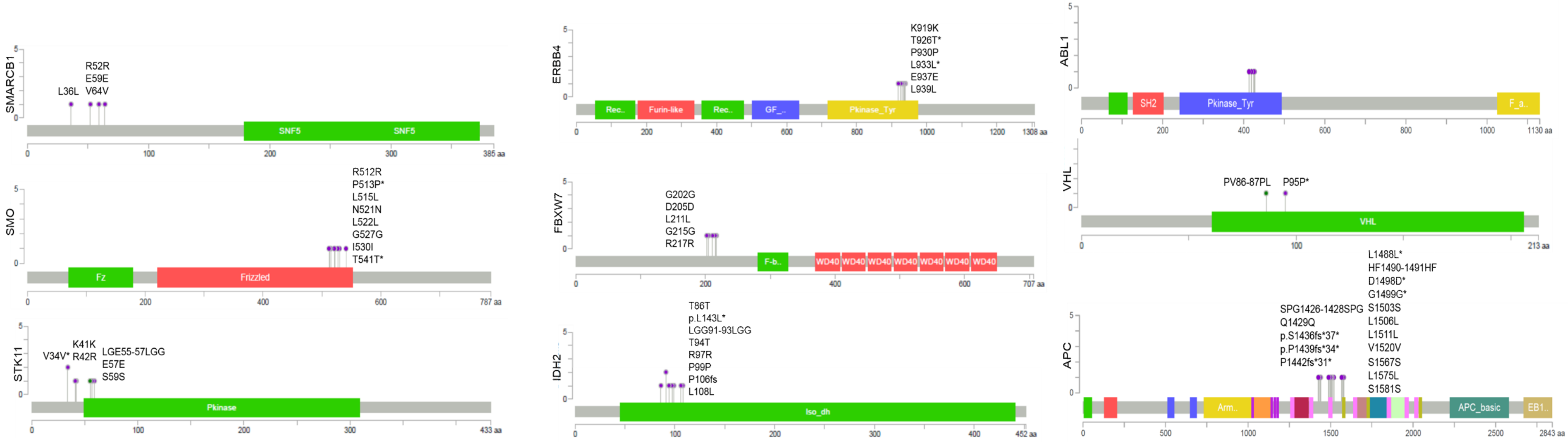

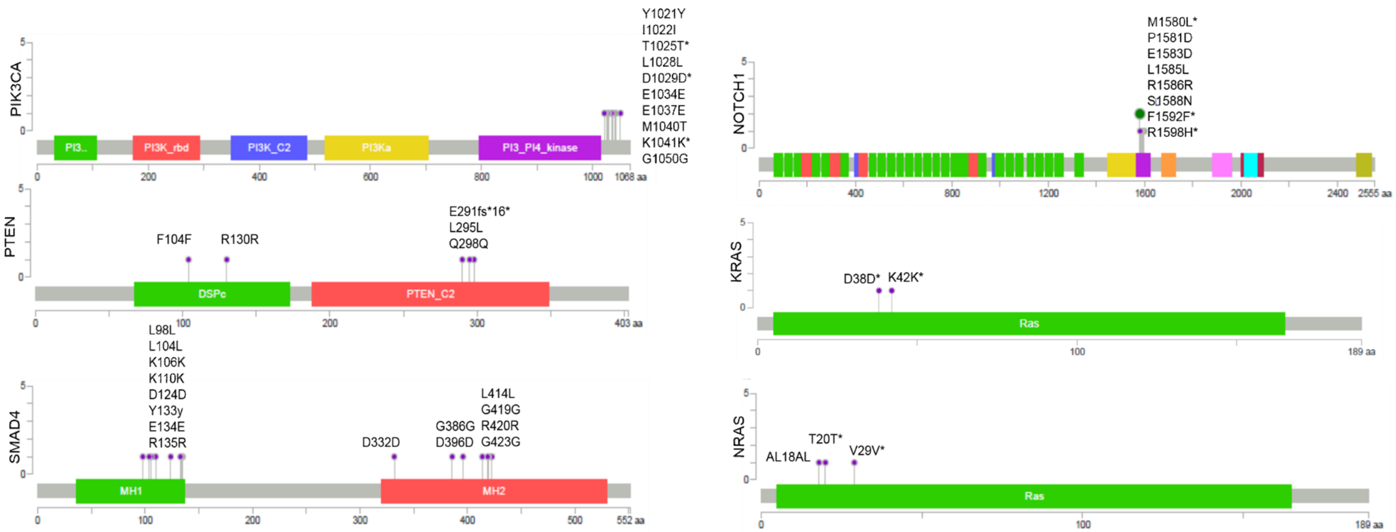

Supplementary figure S2: Identical genomic mutational pattern of CDX model (1-4 generations) derived from sequencing of 50 cancer genes through AmpliSeq Ion Torrent Sequencing panel. Respective gene map showing location and type of mutation present on gene. Green and purple color represents missense and other mutations respectively. Asterisk represents cosmic mutations present in respective gene (http://www.cbioportal.org/mutation_mapper.jsp)

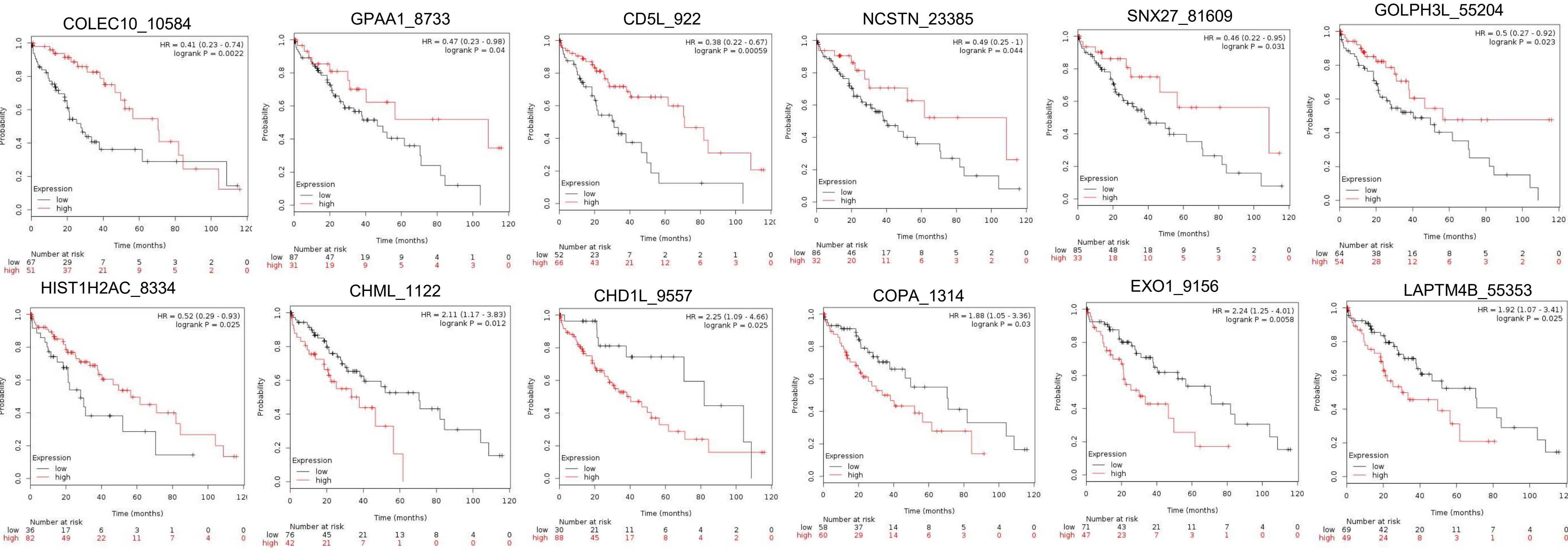

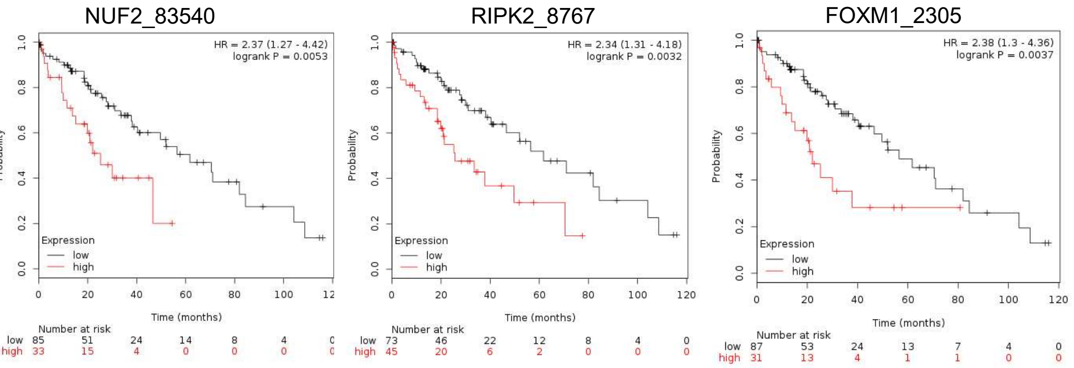

Supplementary figure S3: Survival genes of top 50 genomic altered genes derived from cbioportal analysis. Overall survival analysis were performed in female liver cancer patients using KM-plot. Gene, their selected probe ID, hazard ratio (HR) and logrank *P*-value were mentioned at top of each survival graph. Red indicated high expression and black indicates low expression of respective gene. X-axis representing number of patients survived in months and Y-axis represents the probability.

Supplementary Table S2: Twenty-four gene pairs with significant 502 co-occurrent alterations

Gene A	Gene B	Neither	A Not B	B Not A	Both	Log Odds Ratio	p-Value	Adjusted p-Value	Tendency
SQLE	WASHC5	176	0	0	80	>3	<0.001	<0.001	Co-occurrence
ATAD2	SQLE	173	3	3	77	>3	<0.001	<0.001	Co-occurrence
ATAD2	WASHC5	173	3	3	77	>3	<0.001	<0.001	Co-occurrence
GBA	GBAP1	211	0	0	45	>3	<0.001	<0.001	Co-occurrence
ATP6V1C1	YWHAZ	183	4	4	65	>3	<0.001	<0.001	Co-occurrence
COPA	NCSTN	212	0	0	44	>3	<0.001	<0.001	Co-occurrence
CCT3	SSR2	212	0	0	44	>3	<0.001	<0.001	Co-occurrence
NUF2	RGS5	214	0	0	42	>3	<0.001	<0.001	Co-occurrence
PIP5K1A	PRUNE1	214	0	0	42	>3	<0.001	<0.001	Co-occurrence
CCT3	GBA	211	0	1	44	>3	<0.001	<0.001	Co-occurrence
CCT3	GBAP1	211	0	1	44	>3	<0.001	<0.001	Co-occurrence
COPA	PEA15	211	0	1	44	>3	<0.001	<0.001	Co-occurrence
PEA15	NCSTN	211	1	0	44	>3	<0.001	<0.001	Co-occurrence
GBA	SSR2	211	1	0	44	>3	<0.001	<0.001	Co-occurrence
GBAP1	SSR2	211	1	0	44	>3	<0.001	<0.001	Co-occurrence
ILF2	UBAP2L	208	2	0	46	>3	<0.001	<0.001	Co-occurrence
HIST2H2AC	HIST2H2BE	215	0	0	41	>3	<0.001	<0.001	Co-occurrence
HIST2H2AC	HIST2H4B	215	0	0	41	>3	<0.001	<0.001	Co-occurrence
HIST2H2BE	HIST2H4B	215	0	0	41	>3	<0.001	<0.001	Co-occurrence
COLEC10	ATAD2	166	10	4	76	>3	<0.001	<0.001	Co-occurrence
HIST2H2AA3	HIST2H2BC	216	0	0	40	>3	<0.001	<0.001	Co-occurrence
HIST2H2AA3	HIST2H2AA4	216	0	0	40	>3	<0.001	<0.001	Co-occurrence
HIST2H2BC	HIST2H2AA4	216	0	0	40	>3	<0.001	<0.001	Co-occurrence
GOLPH3L	PIP5K1A	213	1	0	42	>3	<0.001	<0.001	Co-occurrence
GOLPH3L	PRUNE1	213	1	0	42	>3	<0.001	<0.001	Co-occurrence
PEA15	TAGLN2	210	1	1	44	>3	<0.001	<0.001	Co-occurrence
HIST2H2AC	HIST2H2AA3	215	1	0	40	>3	<0.001	<0.001	Co-occurrence
HIST2H2BE	HIST2H2AA3	215	1	0	40	>3	<0.001	<0.001	Co-occurrence
HIST2H4B	HIST2H2AA3	215	1	0	40	>3	<0.001	<0.001	Co-occurrence
HIST2H2AC	HIST2H2BC	215	1	0	40	>3	<0.001	<0.001	Co-occurrence
HIST2H2BE	HIST2H2BC	215	1	0	40	>3	<0.001	<0.001	Co-occurrence
HIST2H4B	HIST2H2BC	215	1	0	40	>3	<0.001	<0.001	Co-occurrence
HIST2H2AC	HIST2H2AA4	215	1	0	40	>3	<0.001	<0.001	Co-occurrence
HIST2H2BE	HIST2H2AA4	215	1	0	40	>3	<0.001	<0.001	Co-occurrence
HIST2H4B	HIST2H2AA4	215	1	0	40	>3	<0.001	<0.001	Co-occurrence
GOLPH3L	VPS45	212	1	1	42	>3	<0.001	<0.001	Co-occurrence
GBA	UBAP2L	209	1	2	44	>3	<0.001	<0.001	Co-occurrence
GBAP1	UBAP2L	209	1	2	44	>3	<0.001	<0.001	Co-occurrence

GNPAT	TTC13	216	0	1	39	>3	<0.001	<0.001	Co-occurrence
COPA	TAGLN2	210	1	2	43	>3	<0.001	<0.001	Co-occurrence
NCSTN	TAGLN2	210	1	2	43	>3	<0.001	<0.001	Co-occurrence
CCT3	CD5L	211	2	1	42	>3	<0.001	<0.001	Co-occurrence
CD5L	SSR2	211	1	2	42	>3	<0.001	<0.001	Co-occurrence
CCT3	UBAP2L	209	1	3	43	>3	<0.001	<0.001	Co-occurrence
SSR2	UBAP2L	209	1	3	43	>3	<0.001	<0.001	Co-occurrence
PIP5K1A	VPS45	212	1	2	41	>3	<0.001	<0.001	Co-occurrence
PRUNE1	VPS45	212	1	2	41	>3	<0.001	<0.001	Co-occurrence
COLEC10	SQLE	164	12	6	74	>3	<0.001	<0.001	Co-occurrence
COLEC10	WASHC5	164	12	6	74	>3	<0.001	<0.001	Co-occurrence
ILF2	GBA	207	4	1	44	>3	<0.001	<0.001	Co-occurrence
ILF2	GBAP1	207	4	1	44	>3	<0.001	<0.001	Co-occurrence
RBM12B	INTS8	198	5	3	50	>3	<0.001	<0.001	Co-occurrence
CD5L	GBA	210	1	3	42	>3	<0.001	<0.001	Co-occurrence
CD5L	GBAP1	210	1	3	42	>3	<0.001	<0.001	Co-occurrence
SNX27	PIP5K1A	211	3	1	41	>3	<0.001	<0.001	Co-occurrence
SNX27	PRUNE1	211	3	1	41	>3	<0.001	<0.001	Co-occurrence
S100A10	SNX27	205	7	0	44	>3	<0.001	<0.001	Co-occurrence
CHML	EXO1	223	0	0	33	>3	<0.001	<0.001	Co-occurrence
ILF2	CCT3	207	5	1	43	>3	<0.001	<0.001	Co-occurrence
ILF2	SSR2	207	5	1	43	>3	<0.001	<0.001	Co-occurrence
SNX27	GOLPH3L	210	3	2	41	>3	<0.001	<0.001	Co-occurrence
RBM12B	RIPK2	196	5	5	50	>3	<0.001	<0.001	Co-occurrence
YWHAZ	LAPTM4B	185	13	2	56	>3	<0.001	<0.001	Co-occurrence
CD5L	TAGLN2	209	2	4	41	>3	<0.001	<0.001	Co-occurrence
GNPAT	SIPA1L2	215	2	2	37	>3	<0.001	<0.001	Co-occurrence
CD5L	UBAP2L	208	2	5	41	>3	<0.001	<0.001	Co-occurrence
CCT3	TAGLN2	208	3	4	41	>3	<0.001	<0.001	Co-occurrence
SSR2	TAGLN2	208	3	4	41	>3	<0.001	<0.001	Co-occurrence
HIST2H2AC	VPS45	211	2	4	39	>3	<0.001	<0.001	Co-occurrence
HIST2H2BE	VPS45	211	2	4	39	>3	<0.001	<0.001	Co-occurrence
HIST2H4B	VPS45	211	2	4	39	>3	<0.001	<0.001	Co-occurrence
ATP6V1C1	LAPTM4B	184	14	3	55	>3	<0.001	<0.001	Co-occurrence
SIPA1L2	TTC13	214	2	3	37	>3	<0.001	<0.001	Co-occurrence
SNX27	VPS45	209	4	3	40	>3	<0.001	<0.001	Co-occurrence
GBA	TAGLN2	207	4	4	41	>3	<0.001	<0.001	Co-occurrence
GBAP1	TAGLN2	207	4	4	41	>3	<0.001	<0.001	Co-occurrence
S100A10	GOLPH3L	204	9	1	42	>3	<0.001	<0.001	Co-occurrence
RIPK2	ZNF704	198	9	3	46	>3	<0.001	<0.001	Co-occurrence
ILF2	CD5L	206	7	2	41	>3	<0.001	<0.001	Co-occurrence
CD5L	PEA15	208	3	5	40	>3	<0.001	<0.001	Co-occurrence

HIST2H2AA3	VPS45	211	2	5	38	>3	<0.001	<0.001	Co-occurrence
HIST2H2BC	VPS45	211	2	5	38	>3	<0.001	<0.001	Co-occurrence
VPS45	HIST2H2AA4	211	5	2	38	>3	<0.001	<0.001	Co-occurrence
HIST2H2AC	PIP5K1A	211	3	4	38	>3	<0.001	<0.001	Co-occurrence
HIST2H2BE	PIP5K1A	211	3	4	38	>3	<0.001	<0.001	Co-occurrence
HIST2H4B	PIP5K1A	211	3	4	38	>3	<0.001	<0.001	Co-occurrence
HIST2H2AC	PRUNE1	211	3	4	38	>3	<0.001	<0.001	Co-occurrence
HIST2H2BE	PRUNE1	211	3	4	38	>3	<0.001	<0.001	Co-occurrence
HIST2H4B	PRUNE1	211	3	4	38	>3	<0.001	<0.001	Co-occurrence
CHD1L	HIST2H2AA3	214	2	4	36	>3	<0.001	<0.001	Co-occurrence
CHD1L	HIST2H2BC	214	2	4	36	>3	<0.001	<0.001	Co-occurrence
CHD1L	HIST2H2AA4	214	2	4	36	>3	<0.001	<0.001	Co-occurrence
S100A10	PIP5K1A	204	10	1	41	>3	<0.001	<0.001	Co-occurrence
S100A10	PRUNE1	204	10	1	41	>3	<0.001	<0.001	Co-occurrence
CCT3	PEA15	207	4	5	40	>3	<0.001	<0.001	Co-occurrence
PEA15	SSR2	207	5	4	40	>3	<0.001	<0.001	Co-occurrence
GOLPH3L	HIST2H2AC	210	5	3	38	>3	<0.001	<0.001	Co-occurrence
GOLPH3L	HIST2H2BE	210	5	3	38	>3	<0.001	<0.001	Co-occurrence
GOLPH3L	HIST2H4B	210	5	3	38	>3	<0.001	<0.001	Co-occurrence
LAPTM4B	INTS8	193	10	5	48	>3	<0.001	<0.001	Co-occurrence
CHD1L	HIST2H2AC	213	2	5	36	>3	<0.001	<0.001	Co-occurrence
CHD1L	HIST2H2BE	213	2	5	36	>3	<0.001	<0.001	Co-occurrence
CHD1L	HIST2H4B	213	2	5	36	>3	<0.001	<0.001	Co-occurrence
CD5L	COPA	208	4	5	39	>3	<0.001	<0.001	Co-occurrence
CD5L	NCSTN	208	4	5	39	>3	<0.001	<0.001	Co-occurrence
GBA	PEA15	206	5	5	40	>3	<0.001	<0.001	Co-occurrence
GBAP1	PEA15	206	5	5	40	>3	<0.001	<0.001	Co-occurrence
HIST2H2AA3	PIP5K1A	211	3	5	37	>3	<0.001	<0.001	Co-occurrence
HIST2H2BC	PIP5K1A	211	3	5	37	>3	<0.001	<0.001	Co-occurrence
HIST2H2AA3	PRUNE1	211	3	5	37	>3	<0.001	<0.001	Co-occurrence
HIST2H2BC	PRUNE1	211	3	5	37	>3	<0.001	<0.001	Co-occurrence
PIP5K1A	HIST2H2AA4	211	5	3	37	>3	<0.001	<0.001	Co-occurrence
PRUNE1	HIST2H2AA4	211	5	3	37	>3	<0.001	<0.001	Co-occurrence
S100A10	VPS45	203	10	2	41	>3	<0.001	<0.001	Co-occurrence
CCT3	COPA	207	5	5	39	>3	<0.001	<0.001	Co-occurrence
CCT3	NCSTN	207	5	5	39	>3	<0.001	<0.001	Co-occurrence
COPA	SSR2	207	5	5	39	>3	<0.001	<0.001	Co-occurrence
NCSTN	SSR2	207	5	5	39	>3	<0.001	<0.001	Co-occurrence
COLEC10	ATP6V1C1	165	22	5	64	>3	<0.001	<0.001	Co-occurrence
GOLPH3L	HIST2H2AA3	210	6	3	37	>3	<0.001	<0.001	Co-occurrence
GOLPH3L	HIST2H2BC	210	6	3	37	>3	<0.001	<0.001	Co-occurrence
GOLPH3L	HIST2H2AA4	210	6	3	37	>3	<0.001	<0.001	Co-occurrence

TAGLN2	UBAP2L	205	5	6	40	>3	<0.001	<0.001	Co-occurrence
LAPTM4B	RBM12B	191	10	7	48	>3	<0.001	<0.001	Co-occurrence
TARBP1	RBM34	214	7	1	34	>3	<0.001	<0.001	Co-occurrence
COPA	GBA	206	5	6	39	>3	<0.001	<0.001	Co-occurrence
COPA	GBAP1	206	5	6	39	>3	<0.001	<0.001	Co-occurrence
GBA	NCSTN	206	6	5	39	>3	<0.001	<0.001	Co-occurrence
GBAP1	NCSTN	206	6	5	39	>3	<0.001	<0.001	Co-occurrence
RIPK2	INTS8	194	9	7	46	>3	<0.001	<0.001	Co-occurrence
PEA15	NUF2	207	7	4	38	>3	<0.001	<0.001	Co-occurrence
PEA15	RGS5	207	7	4	38	>3	<0.001	<0.001	Co-occurrence
NUF2	TAGLN2	207	4	7	38	>3	<0.001	<0.001	Co-occurrence
RGS5	TAGLN2	207	4	7	38	>3	<0.001	<0.001	Co-occurrence
ILF2	TAGLN2	203	8	5	40	>3	<0.001	<0.001	Co-occurrence
COLEC10	YWHAZ	164	23	6	63	>3	<0.001	<0.001	Co-occurrence
SNX27	HIST2H2AC	208	7	4	37	>3	<0.001	<0.001	Co-occurrence
SNX27	HIST2H2BE	208	7	4	37	>3	<0.001	<0.001	Co-occurrence
SNX27	HIST2H4B	208	7	4	37	>3	<0.001	<0.001	Co-occurrence
ATAD2	ATP6V1C1	168	19	8	61	>3	<0.001	<0.001	Co-occurrence
CD5L	NUF2	208	6	5	37	>3	<0.001	<0.001	Co-occurrence
CD5L	RGS5	208	6	5	37	>3	<0.001	<0.001	Co-occurrence
PEA15	UBAP2L	204	6	7	39	>3	<0.001	<0.001	Co-occurrence
MTR	CHML	218	5	2	31	>3	<0.001	<0.001	Co-occurrence
MTR	EXO1	218	5	2	31	>3	<0.001	<0.001	Co-occurrence
CCT3	NUF2	207	7	5	37	>3	<0.001	<0.001	Co-occurrence
COPA	NUF2	207	7	5	37	>3	<0.001	<0.001	Co-occurrence
NCSTN	NUF2	207	7	5	37	>3	<0.001	<0.001	Co-occurrence
CCT3	RGS5	207	7	5	37	>3	<0.001	<0.001	Co-occurrence
COPA	RGS5	207	7	5	37	>3	<0.001	<0.001	Co-occurrence
NCSTN	RGS5	207	7	5	37	>3	<0.001	<0.001	Co-occurrence
SSR2	RGS5	207	7	5	37	>3	<0.001	<0.001	Co-occurrence
NUF2	SSR2	207	5	7	37	>3	<0.001	<0.001	Co-occurrence
CHD1L	VPS45	210	3	8	35	>3	<0.001	<0.001	Co-occurrence
SNX27	HIST2H2AA3	208	8	4	36	>3	<0.001	<0.001	Co-occurrence
SNX27	HIST2H2BC	208	8	4	36	>3	<0.001	<0.001	Co-occurrence
SNX27	HIST2H2AA4	208	8	4	36	>3	<0.001	<0.001	Co-occurrence
ATP6V1C1	INTS8	183	20	4	49	>3	<0.001	<0.001	Co-occurrence
YWHAZ	INTS8	183	20	4	49	>3	<0.001	<0.001	Co-occurrence
GBA	NUF2	206	8	5	37	>3	<0.001	<0.001	Co-occurrence
GBAP1	NUF2	206	8	5	37	>3	<0.001	<0.001	Co-occurrence
GBA	RGS5	206	8	5	37	>3	<0.001	<0.001	Co-occurrence
GBAP1	RGS5	206	8	5	37	>3	<0.001	<0.001	Co-occurrence
SNX27	CHD1L	209	9	3	35	>3	<0.001	<0.001	Co-occurrence

SQLE	ATP6V1C1	167	20	9	60	>3	<0.001	<0.001	Co-occurrence
WASHC5	ATP6V1C1	167	20	9	60	>3	<0.001	<0.001	Co-occurrence
COPA	UBAP2L	204	6	8	38	>3	<0.001	<0.001	Co-occurrence
NCSTN	UBAP2L	204	6	8	38	>3	<0.001	<0.001	Co-occurrence
ILF2	PEA15	202	9	6	39	>3	<0.001	<0.001	Co-occurrence
S100A10	HIST2H2AC	202	13	3	38	>3	<0.001	<0.001	Co-occurrence
S100A10	HIST2H2BE	202	13	3	38	>3	<0.001	<0.001	Co-occurrence
S100A10	HIST2H4B	202	13	3	38	>3	<0.001	<0.001	Co-occurrence
NUF2	UBAP2L	205	5	9	37	>3	<0.001	<0.001	Co-occurrence
RGS5	UBAP2L	205	5	9	37	>3	<0.001	<0.001	Co-occurrence
ZNF704	ARMC1	204	12	3	37	>3	<0.001	<0.001	Co-occurrence
CHD1L	PIP5K1A	210	4	8	34	>3	<0.001	<0.001	Co-occurrence
CHD1L	PRUNE1	210	4	8	34	>3	<0.001	<0.001	Co-occurrence
ILF2	COPA	202	10	6	38	>3	<0.001	<0.001	Co-occurrence
ILF2	NCSTN	202	10	6	38	>3	<0.001	<0.001	Co-occurrence
CHD1L	GOLPH3L	209	4	9	34	>3	<0.001	<0.001	Co-occurrence
S100A10	HIST2H2AA3	202	14	3	37	>3	<0.001	<0.001	Co-occurrence
S100A10	HIST2H2BC	202	14	3	37	>3	<0.001	<0.001	Co-occurrence
S100A10	HIST2H2AA4	202	14	3	37	>3	<0.001	<0.001	Co-occurrence
ATAD2	YWHAZ	166	21	10	59	>3	<0.001	<0.001	Co-occurrence
SQLE	YWHAZ	166	21	10	59	>3	<0.001	<0.001	Co-occurrence
WASHC5	YWHAZ	166	21	10	59	>3	<0.001	<0.001	Co-occurrence
S100A10	CHD1L	203	15	2	36	>3	<0.001	<0.001	Co-occurrence
ILF2	NUF2	203	11	5	37	>3	<0.001	<0.001	Co-occurrence
ILF2	RGS5	203	11	5	37	>3	<0.001	<0.001	Co-occurrence
RBM12B	ZNF704	194	13	7	42	>3	<0.001	<0.001	Co-occurrence
INTS8	ZNF704	195	12	8	41	>3	<0.001	<0.001	Co-occurrence
LAPTM4B	RIPK2	188	13	10	45	>3	<0.001	<0.001	Co-occurrence
MTR	RBM34	215	6	5	30	>3	<0.001	<0.001	Co-occurrence
ATP6V1C1	RBM12B	180	21	7	48	>3	<0.001	<0.001	Co-occurrence
YWHAZ	RBM12B	180	21	7	48	>3	<0.001	<0.001	Co-occurrence
YWHAZ	RIPK2	180	21	7	48	>3	<0.001	<0.001	Co-occurrence
RIPK2	ARMC1	198	18	3	37	>3	<0.001	<0.001	Co-occurrence
ILF2	GOLPH3L	201	12	7	36	>3	<0.001	<0.001	Co-occurrence
ILF2	VPS45	201	12	7	36	>3	<0.001	<0.001	Co-occurrence
GNPAT	RBM34	212	9	5	30	>3	<0.001	<0.001	Co-occurrence
SIPA1L2	RBM34	212	9	5	30	>3	<0.001	<0.001	Co-occurrence
YWHAZ	ZNF704	182	25	5	44	>3	<0.001	<0.001	Co-occurrence
TTC13	RBM34	211	10	5	30	>3	<0.001	<0.001	Co-occurrence
ILF2	PIP5K1A	201	13	7	35	>3	<0.001	<0.001	Co-occurrence
ILF2	PRUNE1	201	13	7	35	>3	<0.001	<0.001	Co-occurrence
ATAD2	LAPTM4B	169	29	7	51	>3	<0.001	<0.001	Co-occurrence

SQLE	LAPTM4B	169	29	7	51	>3	<0.001	<0.001	Co-occurrence
WASHC5	LAPTM4B	169	29	7	51	>3	<0.001	<0.001	Co-occurrence
S100A10	ILF2	195	13	10	38	>3	<0.001	<0.001	Co-occurrence
UBAP2L	PIP5K1A	202	12	8	34	>3	<0.001	<0.001	Co-occurrence
UBAP2L	PRUNE1	202	12	8	34	>3	<0.001	<0.001	Co-occurrence
ATAD2	RBM12B	170	31	6	49	>3	<0.001	<0.001	Co-occurrence
ATP6V1C1	RIPK2	178	23	9	46	>3	<0.001	<0.001	Co-occurrence
GBA	CHD1L	205	13	6	32	>3	<0.001	<0.001	Co-occurrence
GBAP1	CHD1L	205	13	6	32	>3	<0.001	<0.001	Co-occurrence
ATP6V1C1	ZNF704	181	26	6	43	>3	<0.001	<0.001	Co-occurrence
LAPTM4B	ZNF704	189	18	9	40	>3	<0.001	<0.001	Co-occurrence
ILF2	SNX27	199	13	9	35	>3	<0.001	<0.001	Co-occurrence
UBAP2L	GOLPH3L	201	12	9	34	>3	<0.001	<0.001	Co-occurrence
UBAP2L	VPS45	201	12	9	34	>3	<0.001	<0.001	Co-occurrence
TARBP1	GNPAT	207	10	8	31	>3	<0.001	<0.001	Co-occurrence
TARBP1	SIPA1L2	207	10	8	31	>3	<0.001	<0.001	Co-occurrence
GBA	SNX27	201	11	10	34	>3	<0.001	<0.001	Co-occurrence
GBAP1	SNX27	201	11	10	34	>3	<0.001	<0.001	Co-occurrence
RBM34	CHML	215	8	6	27	>3	<0.001	<0.001	Co-occurrence
RBM34	EXO1	215	8	6	27	>3	<0.001	<0.001	Co-occurrence
S100A10	GBA	196	15	9	36	>3	<0.001	<0.001	Co-occurrence
S100A10	GBAP1	196	15	9	36	>3	<0.001	<0.001	Co-occurrence
GBA	PIP5K1A	202	12	9	33	>3	<0.001	<0.001	Co-occurrence
GBAP1	PIP5K1A	202	12	9	33	>3	<0.001	<0.001	Co-occurrence
GBA	PRUNE1	202	12	9	33	>3	<0.001	<0.001	Co-occurrence
GBAP1	PRUNE1	202	12	9	33	>3	<0.001	<0.001	Co-occurrence
SNX27	UBAP2L	200	10	12	34	>3	<0.001	<0.001	Co-occurrence
TARBP1	TTC13	206	10	9	31	>3	<0.001	<0.001	Co-occurrence
ATAD2	INTS8	170	33	6	47	>3	<0.001	<0.001	Co-occurrence
SQLE	RBM12B	169	32	7	48	>3	<0.001	<0.001	Co-occurrence
WASHC5	RBM12B	169	32	7	48	>3	<0.001	<0.001	Co-occurrence
CCT3	CHD1L	205	13	7	31	>3	<0.001	<0.001	Co-occurrence
SSR2	CHD1L	205	13	7	31	>3	<0.001	<0.001	Co-occurrence
GBA	HIST2H2AA3	203	13	8	32	>3	<0.001	<0.001	Co-occurrence
GBAP1	HIST2H2AA3	203	13	8	32	>3	<0.001	<0.001	Co-occurrence
GBA	HIST2H2BC	203	13	8	32	>3	<0.001	<0.001	Co-occurrence
GBAP1	HIST2H2BC	203	13	8	32	>3	<0.001	<0.001	Co-occurrence
GBA	HIST2H2AA4	203	13	8	32	>3	<0.001	<0.001	Co-occurrence
GBAP1	HIST2H2AA4	203	13	8	32	>3	<0.001	<0.001	Co-occurrence
COLEC10	LAPTM4B	163	35	7	51	>3	<0.001	<0.001	Co-occurrence
S100A10	UBAP2L	195	15	10	36	>3	<0.001	<0.001	Co-occurrence
GBA	GOLPH3L	201	12	10	33	>3	<0.001	<0.001	Co-occurrence

GBAP1	GOLPH3L	201	12	10	33	>3	<0.001	<0.001	Co-occurrence
GBA	VPS45	201	12	10	33	>3	<0.001	<0.001	Co-occurrence
GBAP1	VPS45	201	12	10	33	>3	<0.001	<0.001	Co-occurrence
CCT3	SNX27	201	11	11	33	>3	<0.001	<0.001	Co-occurrence
SSR2	SNX27	201	11	11	33	>3	<0.001	<0.001	Co-occurrence
ILF2	HIST2H2AC	200	15	8	33	>3	<0.001	<0.001	Co-occurrence
ILF2	HIST2H2BE	200	15	8	33	>3	<0.001	<0.001	Co-occurrence
ILF2	HIST2H4B	200	15	8	33	>3	<0.001	<0.001	Co-occurrence
S100A10	CCT3	196	16	9	35	>3	<0.001	<0.001	Co-occurrence
S100A10	SSR2	196	16	9	35	>3	<0.001	<0.001	Co-occurrence
UBAP2L	HIST2H2AA3	202	14	8	32	>3	<0.001	<0.001	Co-occurrence
UBAP2L	HIST2H2BC	202	14	8	32	>3	<0.001	<0.001	Co-occurrence
UBAP2L	HIST2H2AA4	202	14	8	32	>3	<0.001	<0.001	Co-occurrence
GBA	HIST2H2AC	202	13	9	32	>3	<0.001	<0.001	Co-occurrence
GBAP1	HIST2H2AC	202	13	9	32	>3	<0.001	<0.001	Co-occurrence
GBA	HIST2H2BE	202	13	9	32	>3	<0.001	<0.001	Co-occurrence
GBAP1	HIST2H2BE	202	13	9	32	>3	<0.001	<0.001	Co-occurrence
GBA	HIST2H4B	202	13	9	32	>3	<0.001	<0.001	Co-occurrence
GBAP1	HIST2H4B	202	13	9	32	>3	<0.001	<0.001	Co-occurrence
TARBP1	MTR	208	12	7	29	>3	<0.001	<0.001	Co-occurrence
CCT3	PIP5K1A	202	12	10	32	>3	<0.001	<0.001	Co-occurrence
SSR2	PIP5K1A	202	12	10	32	>3	<0.001	<0.001	Co-occurrence
CCT3	PRUNE1	202	12	10	32	>3	<0.001	<0.001	Co-occurrence
SSR2	PRUNE1	202	12	10	32	>3	<0.001	<0.001	Co-occurrence
UBAP2L	CHD1L	203	15	7	31	>3	<0.001	<0.001	Co-occurrence
UBAP2L	HIST2H2AC	201	14	9	32	>3	<0.001	<0.001	Co-occurrence
UBAP2L	HIST2H2BE	201	14	9	32	>3	<0.001	<0.001	Co-occurrence
UBAP2L	HIST2H4B	201	14	9	32	>3	<0.001	<0.001	Co-occurrence
CD5L	CHD1L	205	13	8	30	>3	<0.001	<0.001	Co-occurrence
CCT3	HIST2H2AA3	203	13	9	31	>3	<0.001	<0.001	Co-occurrence
SSR2	HIST2H2AA3	203	13	9	31	>3	<0.001	<0.001	Co-occurrence
CCT3	HIST2H2BC	203	13	9	31	>3	<0.001	<0.001	Co-occurrence
SSR2	HIST2H2BC	203	13	9	31	>3	<0.001	<0.001	Co-occurrence
CCT3	HIST2H2AA4	203	13	9	31	>3	<0.001	<0.001	Co-occurrence
SSR2	HIST2H2AA4	203	13	9	31	>3	<0.001	<0.001	Co-occurrence
CCT3	GOLPH3L	201	12	11	32	>3	<0.001	<0.001	Co-occurrence
SSR2	GOLPH3L	201	12	11	32	>3	<0.001	<0.001	Co-occurrence
CCT3	VPS45	201	12	11	32	>3	<0.001	<0.001	Co-occurrence
SSR2	VPS45	201	12	11	32	>3	<0.001	<0.001	Co-occurrence
CD5L	SNX27	201	11	12	32	>3	<0.001	<0.001	Co-occurrence
GNPAT	ACBD3	211	12	6	27	>3	<0.001	<0.001	Co-occurrence
S100A10	CD5L	196	17	9	34	>3	<0.001	<0.001	Co-occurrence

ILF2	HIST2H2AA3	200	16	8	32	>3	<0.001	<0.001	Co-occurrence
ILF2	HIST2H2BC	200	16	8	32	>3	<0.001	<0.001	Co-occurrence
ILF2	HIST2H2AA4	200	16	8	32	>3	<0.001	<0.001	Co-occurrence
GNPAT	MTR	209	11	8	28	>3	<0.001	<0.001	Co-occurrence
SIPA1L2	MTR	209	11	8	28	>3	<0.001	<0.001	Co-occurrence
ILF2	CHD1L	201	17	7	31	>3	<0.001	<0.001	Co-occurrence
CCT3	HIST2H2AC	202	13	10	31	>3	<0.001	<0.001	Co-occurrence
SSR2	HIST2H2AC	202	13	10	31	>3	<0.001	<0.001	Co-occurrence
CCT3	HIST2H2BE	202	13	10	31	>3	<0.001	<0.001	Co-occurrence
SSR2	HIST2H2BE	202	13	10	31	>3	<0.001	<0.001	Co-occurrence
CCT3	HIST2H4B	202	13	10	31	>3	<0.001	<0.001	Co-occurrence
SSR2	HIST2H4B	202	13	10	31	>3	<0.001	<0.001	Co-occurrence
TTC13	ACBD3	210	13	6	27	>3	<0.001	<0.001	Co-occurrence
COLEC10	INTS8	164	39	6	47	>3	<0.001	<0.001	Co-occurrence
INTS8	ARMC1	196	20	7	33	>3	<0.001	<0.001	Co-occurrence
TTC13	MTR	208	12	8	28	>3	<0.001	<0.001	Co-occurrence
PEA15	CHD1L	203	15	8	30	>3	<0.001	<0.001	Co-occurrence
TAGLN2	CHD1L	203	15	8	30	>3	<0.001	<0.001	Co-occurrence
PEA15	SNX27	199	13	12	32	>3	<0.001	<0.001	Co-occurrence
SNX27	TAGLN2	199	12	13	32	>3	<0.001	<0.001	Co-occurrence
NUF2	CHD1L	205	13	9	29	>3	<0.001	<0.001	Co-occurrence
RGS5	CHD1L	205	13	9	29	>3	<0.001	<0.001	Co-occurrence
S100A10	PEA15	194	17	11	34	>3	<0.001	<0.001	Co-occurrence
S100A10	TAGLN2	194	17	11	34	>3	<0.001	<0.001	Co-occurrence
YWHAZ	ARMC1	183	33	4	36	>3	<0.001	<0.001	Co-occurrence
RBM12B	ARMC1	194	22	7	33	>3	<0.001	<0.001	Co-occurrence
SQLE	INTS8	168	35	8	45	>3	<0.001	<0.001	Co-occurrence
WASHC5	INTS8	168	35	8	45	>3	<0.001	<0.001	Co-occurrence
ATAD2	RIPK2	167	34	9	46	>3	<0.001	<0.001	Co-occurrence
SIPA1L2	ACBD3	210	13	7	26	>3	<0.001	<0.001	Co-occurrence
SIPA1L2	CHML	210	13	7	26	>3	<0.001	<0.001	Co-occurrence
SIPA1L2	EXO1	210	13	7	26	>3	<0.001	<0.001	Co-occurrence
COPA	CHD1L	203	15	9	29	>3	<0.001	<0.001	Co-occurrence
NCSTN	CHD1L	203	15	9	29	>3	<0.001	<0.001	Co-occurrence
COPA	SNX27	199	13	13	31	>3	<0.001	<0.001	Co-occurrence
NCSTN	SNX27	199	13	13	31	>3	<0.001	<0.001	Co-occurrence
COLEC10	RBM12B	162	39	8	47	>3	<0.001	<0.001	Co-occurrence
CD5L	PIP5K1A	201	13	12	30	>3	<0.001	<0.001	Co-occurrence
CD5L	PRUNE1	201	13	12	30	>3	<0.001	<0.001	Co-occurrence
S100A10	COPA	194	18	11	33	>3	<0.001	<0.001	Co-occurrence
S100A10	NCSTN	194	18	11	33	>3	<0.001	<0.001	Co-occurrence
ATAD2	GPAA1	160	29	16	51	2.867	<0.001	<0.001	Co-occurrence

LAPTM4B	ARMC1	191	25	7	33	>3	<0.001	<0.001	Co-occurrence
S100A10	NUF2	195	19	10	32	>3	<0.001	<0.001	Co-occurrence
S100A10	RGS5	195	19	10	32	>3	<0.001	<0.001	Co-occurrence
NUF2	SNX27	200	12	14	30	>3	<0.001	<0.001	Co-occurrence
RGS5	SNX27	200	12	14	30	>3	<0.001	<0.001	Co-occurrence
CD5L	GOLPH3L	200	13	13	30	>3	<0.001	<0.001	Co-occurrence
CD5L	VPS45	200	13	13	30	>3	<0.001	<0.001	Co-occurrence
CD5L	HIST2H2AA3	202	14	11	29	>3	<0.001	<0.001	Co-occurrence
CD5L	HIST2H2BC	202	14	11	29	>3	<0.001	<0.001	Co-occurrence
CD5L	HIST2H2AA4	202	14	11	29	>3	<0.001	<0.001	Co-occurrence
TARBP1	CHML	208	15	7	26	>3	<0.001	<0.001	Co-occurrence
TARBP1	EXO1	208	15	7	26	>3	<0.001	<0.001	Co-occurrence
ATAD2	ZNF704	169	38	7	42	>3	<0.001	<0.001	Co-occurrence
SQLE	RIPK2	166	35	10	45	>3	<0.001	<0.001	Co-occurrence
WASHC5	RIPK2	166	35	10	45	>3	<0.001	<0.001	Co-occurrence
ATP6V1C1	ARMC1	182	34	5	35	>3	<0.001	<0.001	Co-occurrence
PEA15	PIP5K1A	199	15	12	30	>3	<0.001	<0.001	Co-occurrence
TAGLN2	PIP5K1A	199	15	12	30	>3	<0.001	<0.001	Co-occurrence
PEA15	PRUNE1	199	15	12	30	>3	<0.001	<0.001	Co-occurrence
TAGLN2	PRUNE1	199	15	12	30	>3	<0.001	<0.001	Co-occurrence
CD5L	HIST2H2AC	201	14	12	29	>3	<0.001	<0.001	Co-occurrence
CD5L	HIST2H2BE	201	14	12	29	>3	<0.001	<0.001	Co-occurrence
CD5L	HIST2H4B	201	14	12	29	>3	<0.001	<0.001	Co-occurrence
NUF2	PIP5K1A	201	13	13	29	>3	<0.001	<0.001	Co-occurrence
RGS5	PIP5K1A	201	13	13	29	>3	<0.001	<0.001	Co-occurrence
NUF2	PRUNE1	201	13	13	29	>3	<0.001	<0.001	Co-occurrence
RGS5	PRUNE1	201	13	13	29	>3	<0.001	<0.001	Co-occurrence
COLEC10	RIPK2	161	40	9	46	>3	<0.001	<0.001	Co-occurrence
SQLE	GPAA1	159	30	17	50	2.747	<0.001	<0.001	Co-occurrence
WASHC5	GPAA1	159	30	17	50	2.747	<0.001	<0.001	Co-occurrence
GNPAT	CHML	209	14	8	25	>3	<0.001	<0.001	Co-occurrence
GNPAT	EXO1	209	14	8	25	>3	<0.001	<0.001	Co-occurrence
PEA15	HIST2H2AA3	200	16	11	29	>3	<0.001	<0.001	Co-occurrence
TAGLN2	HIST2H2AA3	200	16	11	29	>3	<0.001	<0.001	Co-occurrence
PEA15	HIST2H2BC	200	16	11	29	>3	<0.001	<0.001	Co-occurrence
TAGLN2	HIST2H2BC	200	16	11	29	>3	<0.001	<0.001	Co-occurrence
PEA15	HIST2H2AA4	200	16	11	29	>3	<0.001	<0.001	Co-occurrence
TAGLN2	HIST2H2AA4	200	16	11	29	>3	<0.001	<0.001	Co-occurrence
PEA15	GOLPH3L	198	15	13	30	>3	<0.001	<0.001	Co-occurrence
TAGLN2	GOLPH3L	198	15	13	30	>3	<0.001	<0.001	Co-occurrence
PEA15	VPS45	198	15	13	30	>3	<0.001	<0.001	Co-occurrence
TAGLN2	VPS45	198	15	13	30	>3	<0.001	<0.001	Co-occurrence

NUF2	GOLPH3L	200	13	14	29	>3	<0.001	<0.001	Co-occurrence
RGS5	GOLPH3L	200	13	14	29	>3	<0.001	<0.001	Co-occurrence
NUF2	VPS45	200	13	14	29	>3	<0.001	<0.001	Co-occurrence
RGS5	VPS45	200	13	14	29	>3	<0.001	<0.001	Co-occurrence
TTC13	CHML	208	15	8	25	>3	<0.001	<0.001	Co-occurrence
TTC13	EXO1	208	15	8	25	>3	<0.001	<0.001	Co-occurrence
PEA15	HIST2H2AC	199	16	12	29	>3	<0.001	<0.001	Co-occurrence
TAGLN2	HIST2H2AC	199	16	12	29	>3	<0.001	<0.001	Co-occurrence
PEA15	HIST2H2BE	199	16	12	29	>3	<0.001	<0.001	Co-occurrence
TAGLN2	HIST2H2BE	199	16	12	29	>3	<0.001	<0.001	Co-occurrence
PEA15	HIST2H4B	199	16	12	29	>3	<0.001	<0.001	Co-occurrence
TAGLN2	HIST2H4B	199	16	12	29	>3	<0.001	<0.001	Co-occurrence
COPA	PIP5K1A	199	15	13	29	>3	<0.001	<0.001	Co-occurrence
NCSTN	PIP5K1A	199	15	13	29	>3	<0.001	<0.001	Co-occurrence
COPA	PRUNE1	199	15	13	29	>3	<0.001	<0.001	Co-occurrence
NCSTN	PRUNE1	199	15	13	29	>3	<0.001	<0.001	Co-occurrence
COLEC10	GPAA1	154	35	16	51	2.641	<0.001	<0.001	Co-occurrence
COLEC10	ZNF704	163	44	7	42	>3	<0.001	<0.001	Co-occurrence
COPA	GOLPH3L	198	15	14	29	>3	<0.001	<0.001	Co-occurrence
NCSTN	GOLPH3L	198	15	14	29	>3	<0.001	<0.001	Co-occurrence
COPA	VPS45	198	15	14	29	>3	<0.001	<0.001	Co-occurrence
NCSTN	VPS45	198	15	14	29	>3	<0.001	<0.001	Co-occurrence
COPA	HIST2H2AA3	200	16	12	28	>3	<0.001	<0.001	Co-occurrence
NCSTN	HIST2H2AA3	200	16	12	28	>3	<0.001	<0.001	Co-occurrence
COPA	HIST2H2BC	200	16	12	28	>3	<0.001	<0.001	Co-occurrence
NCSTN	HIST2H2BC	200	16	12	28	>3	<0.001	<0.001	Co-occurrence
COPA	HIST2H2AA4	200	16	12	28	>3	<0.001	<0.001	Co-occurrence
NCSTN	HIST2H2AA4	200	16	12	28	>3	<0.001	<0.001	Co-occurrence
ATP6V1C1	GPAA1	165	24	22	45	2.644	<0.001	<0.001	Co-occurrence
GPAA1	YWHAZ	165	22	24	45	2.644	<0.001	<0.001	Co-occurrence
COPA	HIST2H2AC	199	16	13	28	>3	<0.001	<0.001	Co-occurrence
NCSTN	HIST2H2AC	199	16	13	28	>3	<0.001	<0.001	Co-occurrence
COPA	HIST2H2BE	199	16	13	28	>3	<0.001	<0.001	Co-occurrence
NCSTN	HIST2H2BE	199	16	13	28	>3	<0.001	<0.001	Co-occurrence
COPA	HIST2H4B	199	16	13	28	>3	<0.001	<0.001	Co-occurrence
NCSTN	HIST2H4B	199	16	13	28	>3	<0.001	<0.001	Co-occurrence
NUF2	HIST2H2AA3	201	15	13	27	>3	<0.001	<0.001	Co-occurrence
RGS5	HIST2H2AA3	201	15	13	27	>3	<0.001	<0.001	Co-occurrence
NUF2	HIST2H2BC	201	15	13	27	>3	<0.001	<0.001	Co-occurrence
RGS5	HIST2H2BC	201	15	13	27	>3	<0.001	<0.001	Co-occurrence
NUF2	HIST2H2AA4	201	15	13	27	>3	<0.001	<0.001	Co-occurrence
RGS5	HIST2H2AA4	201	15	13	27	>3	<0.001	<0.001	Co-occurrence

SQLE	ZNF704	167	40	9	40	2.921	<0.001	<0.001	Co-occurrence
WASHC5	ZNF704	167	40	9	40	2.921	<0.001	<0.001	Co-occurrence
MTR	ACBD3	210	13	10	23	>3	<0.001	<0.001	Co-occurrence
NUF2	HIST2H2AC	200	15	14	27	>3	<0.001	<0.001	Co-occurrence
RGS5	HIST2H2AC	200	15	14	27	>3	<0.001	<0.001	Co-occurrence
NUF2	HIST2H2BE	200	15	14	27	>3	<0.001	<0.001	Co-occurrence
RGS5	HIST2H2BE	200	15	14	27	>3	<0.001	<0.001	Co-occurrence
NUF2	HIST2H4B	200	15	14	27	>3	<0.001	<0.001	Co-occurrence
RGS5	HIST2H4B	200	15	14	27	>3	<0.001	<0.001	Co-occurrence
COLEC10	ARMC1	166	50	4	36	>3	<0.001	<0.001	Co-occurrence
GPAA1	LAPTM4B	171	27	18	40	2.644	<0.001	<0.001	Co-occurrence
RBM34	ACBD3	210	13	11	22	>3	<0.001	<0.001	Co-occurrence
ATAD2	ARMC1	170	46	6	34	>3	<0.001	<0.001	Co-occurrence
GPAA1	INTS8	173	30	16	37	2.59	<0.001	<0.001	Co-occurrence
GPAA1	RBM12B	171	30	18	37	2.461	<0.001	<0.001	Co-occurrence
SQLE	ARMC1	169	47	7	33	2.83	<0.001	<0.001	Co-occurrence
WASHC5	ARMC1	169	47	7	33	2.83	<0.001	<0.001	Co-occurrence
TARBP1	ACBD3	204	19	11	22	>3	<0.001	<0.001	Co-occurrence
GPAA1	ZNF704	174	33	15	34	2.481	<0.001	<0.001	Co-occurrence
GPAA1	RIPK2	170	31	19	36	2.341	<0.001	<0.001	Co-occurrence
ACBD3	CHML	209	14	14	19	>3	<0.001	<0.001	Co-occurrence
ACBD3	EXO1	209	14	14	19	>3	<0.001	<0.001	Co-occurrence
GPAA1	ARMC1	176	40	13	27	2.212	<0.001	<0.001	Co-occurrence
CD5L	GNPAT	194	23	19	20	2.184	<0.001	<0.001	Co-occurrence
CD5L	TTC13	193	23	20	20	2.127	<0.001	<0.001	Co-occurrence
PEA15	GNPAT	192	25	19	20	2.09	<0.001	<0.001	Co-occurrence
TAGLN2	GNPAT	192	25	19	20	2.09	<0.001	<0.001	Co-occurrence
PEA15	TTC13	191	25	20	20	2.033	<0.001	<0.001	Co-occurrence
TAGLN2	TTC13	191	25	20	20	2.033	<0.001	<0.001	Co-occurrence
CCT3	GNPAT	192	25	20	19	1.987	<0.001	<0.001	Co-occurrence
COPA	GNPAT	192	25	20	19	1.987	<0.001	<0.001	Co-occurrence
NCSTN	GNPAT	192	25	20	19	1.987	<0.001	<0.001	Co-occurrence
SSR2	GNPAT	192	25	20	19	1.987	<0.001	<0.001	Co-occurrence
CD5L	ACBD3	197	26	16	17	2.086	<0.001	<0.001	Co-occurrence
GBA	GNPAT	191	26	20	19	1.943	<0.001	<0.001	Co-occurrence
GBAP1	GNPAT	191	26	20	19	1.943	<0.001	<0.001	Co-occurrence
CCT3	TTC13	191	25	21	19	1.933	<0.001	<0.001	Co-occurrence
COPA	TTC13	191	25	21	19	1.933	<0.001	<0.001	Co-occurrence
NCSTN	TTC13	191	25	21	19	1.933	<0.001	<0.001	Co-occurrence
SSR2	TTC13	191	25	21	19	1.933	<0.001	<0.001	Co-occurrence
CD5L	TARBP1	191	24	22	19	1.928	<0.001	<0.001	Co-occurrence
GBA	TTC13	190	26	21	19	1.889	<0.001	0.001	Co-occurrence

GBAP1	TTC13	190	26	21	19	1.889	<0.001	0.001	Co-occurrence
NUF2	GNPAT	193	24	21	18	1.93	<0.001	0.002	Co-occurrence
RGS5	GNPAT	193	24	21	18	1.93	<0.001	0.002	Co-occurrence
CD5L	SIPA1L2	192	25	21	18	1.884	<0.001	0.002	Co-occurrence
NUF2	TTC13	192	24	22	18	1.879	<0.001	0.003	Co-occurrence
RGS5	TTC13	192	24	22	18	1.879	<0.001	0.003	Co-occurrence
NUF2	ACBD3	197	26	17	16	1.964	<0.001	0.004	Co-occurrence
RGS5	ACBD3	197	26	17	16	1.964	<0.001	0.004	Co-occurrence
CHD1L	ACBD3	200	23	18	15	1.981	<0.001	0.005	Co-occurrence
PEA15	SIPA1L2	190	27	21	18	1.797	<0.001	0.006	Co-occurrence
TAGLN2	SIPA1L2	190	27	21	18	1.797	<0.001	0.006	Co-occurrence
CCT3	ACBD3	195	28	17	16	1.88	<0.001	0.008	Co-occurrence
SSR2	ACBD3	195	28	17	16	1.88	<0.001	0.008	Co-occurrence
UBAP2L	GNPAT	189	28	21	18	1.755	<0.001	0.009	Co-occurrence
CCT3	TARBP1	189	26	23	18	1.739	<0.001	0.01	Co-occurrence
SSR2	TARBP1	189	26	23	18	1.739	<0.001	0.01	Co-occurrence
NUF2	SIPA1L2	192	25	22	17	1.781	<0.001	0.01	Co-occurrence
RGS5	SIPA1L2	192	25	22	17	1.781	<0.001	0.01	Co-occurrence
GBA	ACBD3	194	29	17	16	1.84	<0.001	0.011	Co-occurrence
GBAP1	ACBD3	194	29	17	16	1.84	<0.001	0.011	Co-occurrence
PEA15	ACBD3	194	29	17	16	1.84	<0.001	0.011	Co-occurrence
TAGLN2	ACBD3	194	29	17	16	1.84	<0.001	0.011	Co-occurrence
CHD1L	GNPAT	195	22	23	16	1.819	<0.001	0.012	Co-occurrence
UBAP2L	TTC13	188	28	22	18	1.704	<0.001	0.014	Co-occurrence
GBA	TARBP1	188	27	23	18	1.695	<0.001	0.015	Co-occurrence
GBAP1	TARBP1	188	27	23	18	1.695	<0.001	0.015	Co-occurrence
PEA15	TARBP1	188	27	23	18	1.695	<0.001	0.015	Co-occurrence
TAGLN2	TARBP1	188	27	23	18	1.695	<0.001	0.015	Co-occurrence
CHD1L	TTC13	194	22	24	16	1.771	<0.001	0.018	Co-occurrence
ILF2	GNPAT	187	30	21	18	1.676	<0.001	0.018	Co-occurrence
UBAP2L	TARBP1	187	28	23	18	1.654	<0.001	0.022	Co-occurrence
CCT3	SIPA1L2	190	27	22	17	1.693	<0.001	0.023	Co-occurrence
COPA	SIPA1L2	190	27	22	17	1.693	<0.001	0.023	Co-occurrence
NCSTN	SIPA1L2	190	27	22	17	1.693	<0.001	0.023	Co-occurrence
SSR2	SIPA1L2	190	27	22	17	1.693	<0.001	0.023	Co-occurrence
NUF2	TARBP1	190	25	24	17	1.683	<0.001	0.024	Co-occurrence
RGS5	TARBP1	190	25	24	17	1.683	<0.001	0.024	Co-occurrence
ILF2	TTC13	186	30	22	18	1.624	<0.001	0.029	Co-occurrence
GBA	SIPA1L2	189	28	22	17	1.652	<0.001	0.033	Co-occurrence
GBAP1	SIPA1L2	189	28	22	17	1.652	<0.001	0.033	Co-occurrence
S100A10	IDI1	182	32	23	19	1.547	<0.001	0.041	Co-occurrence
ILF2	TARBP1	185	30	23	18	1.574	<0.001	0.045	Co-occurrence

COPA	ACBD3	194	29	18	15	1.718	<0.001	0.047	Co-occurrence
NCSTN	ACBD3	194	29	18	15	1.718	<0.001	0.047	Co-occurrence
